# Extracellular CIRP induces macrophage endotoxin tolerance through IL-6R–mediated STAT3 activation

**DOI:** 10.1172/jci.insight.133715

**Published:** 2020-03-12

**Authors:** Mian Zhou, Monowar Aziz, Naomi-Liza Denning, Hao-Ting Yen, Gaifeng Ma, Ping Wang

**Affiliations:** 1 Center for Immunology and Inflammation, the Feinstein Institutes for Medical Research, Manhasset, New York, USA.; 2 Elmezzi Graduate School of Molecular Medicine, Manhasset, New York, USA.; 3 Department of Surgery, Donald and Barbara Zucker School of Medicine at Hofstra/Northwell, Manhasset, New York, USA.

**Keywords:** Immunology, Inflammation, Cellular immune response, Innate immunity, Signal transduction

## Abstract

Extracellular cold-inducible RNA-binding protein (eCIRP) is a damage-associated molecular pattern, whose effect on macrophages is not entirely elucidated. Here we identified that eCIRP promotes macrophage endotoxin tolerance. Septic mice had higher serum levels of eCIRP; this was associated with a reduced ex vivo immune response of their splenocytes to LPS. Pretreatment of macrophages with recombinant murine CIRP (rmCIRP) resulted in a tolerance to LPS stimulation as demonstrated by a reduction of TNF-α production. We found that eCIRP increased phosphorylated STAT3 (p-STAT3) in macrophages. A STAT3 inhibitor, Stattic, rescued macrophages from rmCIRP-induced tolerance by restoring the release of TNF-α in response to LPS stimulation. We discovered strong binding affinity between eCIRP and IL-6 receptor (IL-6R) as revealed by Biacore, fluorescence resonance energy transfer (FRET), and their colocalization in macrophages by immunostaining assays. Blockade of IL-6R with its neutralizing Ab inhibited eCIRP-induced p-STAT3 and restored LPS-stimulated TNF-α release in macrophages. Incubation of macrophages with rmCIRP skewed them toward an M2 phenotype, while treatment with anti–IL-6R Ab prevented rmCIRP-induced M2 polarization. Thus, we have demonstrated that eCIRP activates p-STAT3 via a novel receptor, IL-6R, to promote macrophage endotoxin tolerance. Targeting eCIRP appears to be a new therapeutic option to correct immune tolerance in sepsis.

## Abstract 2

Extracellular cold-inducible RNA-binding protein induces macrophage endotoxin tolerance by activating STAT3 through receptor IL-6R.

## Graphical abstract

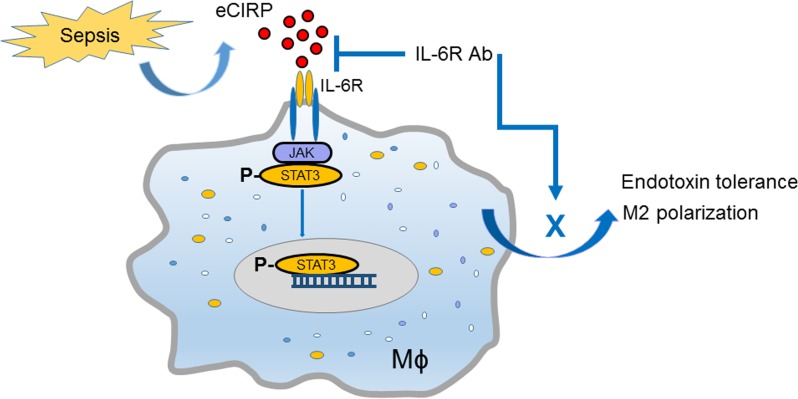

## Introduction

Macrophage “endotoxin tolerance” is defined as a state of LPS hyporesponsiveness, in which macrophages preexposed to endotoxin produce decreased levels of inflammatory mediators upon restimulation with LPS (1, 2). Endotoxin tolerance serves as an important regulatory mechanism to control excessive inflammation. However, prolonged immune tolerance allows the development of secondary infections, increasing morbidity and mortality from sepsis, trauma, and ischemia/reperfusion (I/R) injury (3, 4).

Tolerant macrophages produce lower levels of the inflammatory cytokine tumor necrosis factor–α (TNF-α) and increased levels of the antiinflammatory cytokines IL-10 and transforming growth factor–β (TGF-β) as compared with their nonsensitized counterparts (1, 2, 5–8). Macrophage endotoxin tolerance has been associated with decreased Toll-like receptor 4–myeloid differentiation factor 88 (TLR4-MyD88) complex formation (9), defects in the activation of mitogen-activated protein kinases (MAPKs) and NF-κB (1, 10), and the upregulation of negative regulators like IL-1 receptor associated kinase-M (IRAK-M) (11), ST2 (12), and suppressor of cytokine signaling 1 (SOCS1) (13).

M2 macrophages, like endotoxin-tolerant macrophages, also produce fewer proinflammatory cytokines (e.g., IL-12, TNF-α) and more antiinflammatory cytokines (e.g., IL-10) (1, 14, 15). The STAT3 pathway plays a pivotal role in both macrophage M2 polarization and macrophage immune tolerance (16, 17). IL-6, a pleiotropic cytokine, is produced mainly in macrophages and lymphocytes (18). IL-6 binds to the IL-6 receptor (IL-6R), which is composed of 2 membrane-associated proteins: an 80-kDa α unit for binding IL-6 and 130-kDa β unit (also known as gp130) for downstream signal transduction to activate JAK and the phosphorylation of STAT3 (18). STAT3 activation consequently regulates inflammatory cascades and also can promote M2 polarization (16, 19, 20). Because M2 macrophages resemble endotoxin-tolerant macrophages, the involvement of IL-6R/STAT3 signaling in macrophage endotoxin tolerance is logical.

The mechanisms of macrophage endotoxin tolerance have mainly been elucidated in LPS-TLR4–mediated endotoxin tolerance or homotolerance. However, endotoxin tolerance can also develop upon preexposure of macrophages to TLR2 ligands (e.g., lipoteichoic acid), a phenomenon known as cross-tolerance (21–23), or from chronic exposure to TNF-α and IL-1β (21). Although damage-associated molecular patterns (DAMPs) have been shown to induce tolerance in macrophages (24), the concept and mechanism of DAMP-mediated endotoxin tolerance is less well described.

Cold-inducible RNA-binding protein (CIRP) is an 18-kDa RNA chaperone that regulates the translation of stress response genes intracellularly (25). CIRP is expressed in numerous cell types, including macrophages, neutrophils, and epithelial and endothelial cells (reviewed in ref. 26). Hypoxia, sepsis, and hemorrhagic shock (HS) can induce the release of CIRP into the extracellular space (26, 27). CIRP is released from macrophages and other cells actively by the lysosomal exocytosis pathway and passively by cellular necrosis (26, 27). Extracellular CIRP (eCIRP) is a new DAMP that fuels inflammation and organ injury in sepsis, HS, and organ I/R (26, 27). Increased serum levels of eCIRP correlate with sepsis severity in patients (27, 28). In macrophages, eCIRP induces inflammation by binding to its receptor, TLR4 (26). However, akin to other DAMPs (29, 30), eCIRP may have numerous receptors other than TLR4 to stimulate immune responses. eCIRP-mediated inflammation through TLR4 has been well studied, while its role in promoting macrophages’ immune tolerance has not been explored. We therefore aimed to study the effects of eCIRP on macrophage endotoxin tolerance and define potential mechanisms for this phenomenon.

We found that pretreatment of macrophages with recombinant CIRP significantly decreased their responsiveness to subsequent LPS stimulation. We revealed that eCIRP-induced macrophage immune tolerance was associated with the activation of STAT3. IL-6R and JAK are often used to initiate STAT3 activation (18). We identified high binding affinity between eCIRP and IL-6R and demonstrated that this binding resulted in STAT3 activation, promoting immune tolerance in macrophages. Thus, we have identified a potentially new mechanism for eCIRP’s deleterious effects in inflammation: the induction of macrophage endotoxin tolerance through IL-6R–mediated STAT3 activation. This finding identifies a new therapeutic target to prevent sepsis-mediated immunosuppression.

## Results

### eCIRP promotes macrophage endotoxin tolerance.

**Figure 1:**
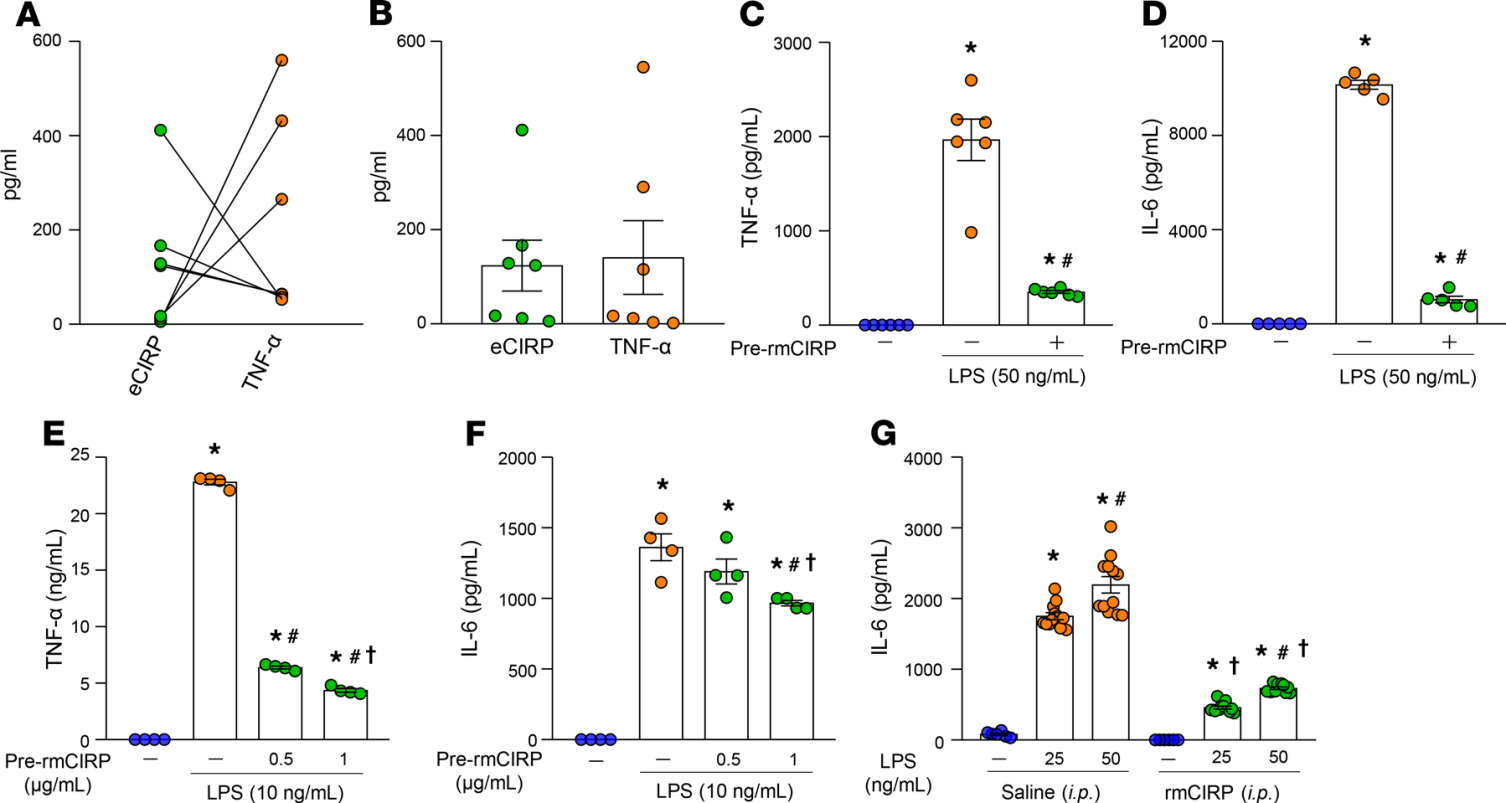
eCIRP induces macrophage tolerance. (**A**) Sepsis was induced in mice by CLP. Blood and spleen were collected 72 hours after CLP. eCIRP levels in the serum were assessed. Splenocytes were isolated from the septic mouse and stimulated with LPS (100 ng/mL) for 5 hours ex vivo and assessed for TNF-α in the culture supernatants. Data show the correlation between serum level of eCIRP and culture supernatant level of TNF-α from splenocytes treated with ex vivo LPS for each mouse. Green circle, eCIRP; orange circle, TNF-α. *n* = 7 mice/group. (**B**) TNF-α levels in the serum were assessed and presented with serum levels of eCIRP. Data are expressed as mean ± SEM (*n* = 7 mice/group). (**C** and **D**) A total of 7 × 10^5^/mL peritoneal macrophages isolated from healthy mice were prestimulated with PBS or rmCIRP (1 μg/mL) for 24 hours, and the cells were washed with medium. Macrophages were restimulated with LPS (50 ng/mL) for 5 hours and assessed for (**C**) TNF-α and (**D**) IL-6 in the culture supernatants. Data are expressed as mean ± SEM (*n* = 5–6 wells/group). Experiments were repeated, and the repeated experimental data are shown in Supplemental Figure 7. *P < 0.05 vs. PBS control; ^#^*P* < 0.05 vs. pre-rmCIRP (–), LPS (+). (**E** and **F**) RAW264.7 macrophages (3 × 10^5^/mL) were pretreated with PBS or rmCIRP at 0.5 and 1.0 μg/mL for 24 hours. Cells were washed with medium, restimulated with LPS (10 ng/mL) for 5 hours and assessed for (**E**) TNF-α and (**F**) IL-6 in the culture supernatants. Data are expressed as mean ± SEM (*n* = 4 wells/group). Experiments were repeated, and the repeated experimental data are shown in Supplemental Figure 7. *P < 0.05 vs. PBS control; ^#^*P* < 0.05 vs. pre-rmCIRP (–), LPS (+); ^†^*P* < 0.05 vs. rmCIRP (0.5 μg/mL). (**G**) Mice were injected with normal saline or rmCIRP (5 mg/kg BW) intraperitoneally (i.p.); 24 hours after injection, peritoneal macrophages were isolated. A total of 2 × 10^5^ peritoneal macrophages were stimulated with 25 and 50 ng/mL LPS for 5 hours ex vivo and assessed for IL-6 in the culture supernatants. Data are expressed as mean ± SEM (*n* = 6–12 samples/group). Experiments were performed 2 times, and all data were used for analysis. The groups were compared by 1-way ANOVA and Student-Newman-Keuls (SNK) method. *P < 0.05 vs. PBS in respective injection group, ^#^*P* < 0.05 vs. LPS (25 ng/mL) in respective injection group, and ^†^*P* < 0.05 vs. saline injection in respective LPS dose. CLP, cecal ligation and puncture.

Murine polymicrobial sepsis is bimodal, with an early hyperdynamic phase (2–10 hours after cecal ligation and puncture [CLP]) characterized by an overwhelming inflammatory response, followed by a late hypodynamic phase (20 hours after CLP) resulting in immunosuppression or tolerance (31–34). We assessed serum levels of eCIRP in septic mice 72 hours after CLP and correlated the results with the amount of TNF-α produced by splenocytes isolated from the spleens of the same mice after ex vivo LPS stimulation. Interestingly, we found that the higher serum levels of eCIRP after CLP correlated with decreased TNF-α production by splenocytes after ex vivo LPS stimulation (Figure 1A).

Correspondingly, splenocytes isolated from mice with lower serum levels of eCIRP produced higher levels of TNF-α upon stimulation with LPS (Figure 1A). We also assessed their serum levels of TNF-α and found they were parallel to serum levels of eCIRP (Figure 1B).

We performed an in vitro experiment by pretreating peritoneal macrophages isolated from healthy mice with either PBS or recombinant murine (rm) CIRP for 24 hours, and then we restimulated these cells with LPS for 5 hours. We found that peritoneal macrophages pretreated with rmCIRP produced significantly decreased levels of TNF-α and IL-6 by 82% and 90%, respectively, in the culture supernatants compared with cells untreated with rmCIRP (Figure 1, C and D). A similar finding was obtained in the macrophage cell line RAW264.7, in which pretreatment with various doses of rmCIRP resulted in significantly decreased production of TNF-α and IL-6 in the supernatants in a dose-dependent manner, compared with pre-rmCIRP–untreated controls after stimulation with a fixed dose of LPS (Figure 1, E and F). Next, in an in vivo study, we injected mice with rmCIRP i.p., isolated peritoneal macrophages 24 hours later, and stimulated with LPS ex vivo for 5 hours. We found that the peritoneal macrophages isolated from rmCIRP-injected mice produced significantly decreased levels of IL-6 by 74% and 67% at 25 and 50 ng/mL of LPS stimulation, respectively, compared with macrophages isolated from saline-injected mice (Figure 1G). Collectively, the results of these in vitro, ex vivo, and in vivo studies confirm eCIRP’s ability to induce immune tolerance in macrophages.

### STAT3 is activated in eCIRP-treated macrophages.

**Figure 2:**
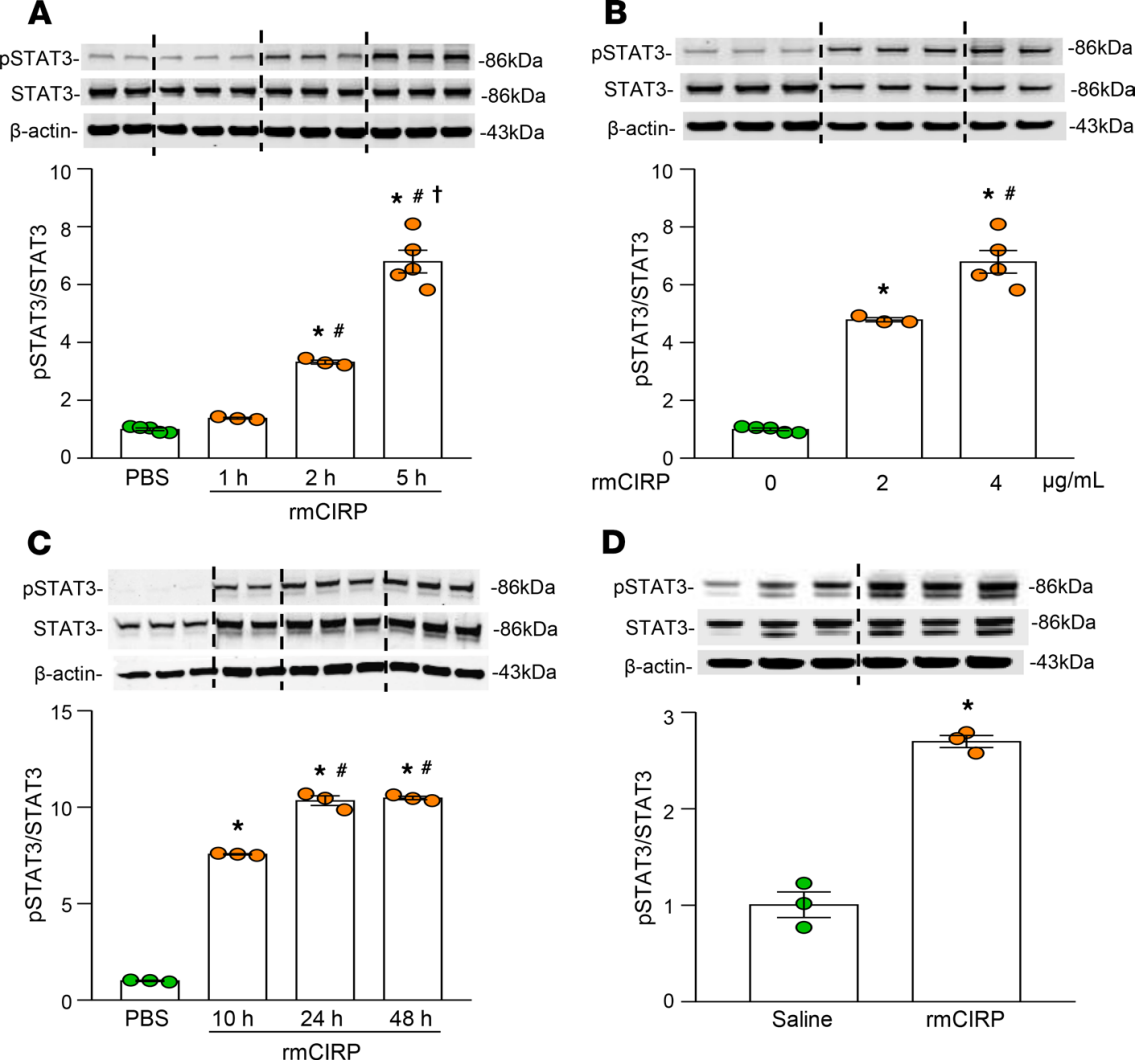
eCIRP induces STAT3 phosphorylation in macrophages. (**A**) RAW264.7 cells (8 × 10^5^ cells/mL) were stimulated with rmCIRP (2 μg/mL) for 1, 2, and 5 hours. Cells were harvested for protein extraction, followed by Western blot using Abs against p-STAT3, STAT3, and β-actin. Data are expressed as mean ± SEM (*n* = 3–5 samples/group). Experiments were repeated, and the repeated experimental data are shown in Supplemental Figure 8. *P < 0.05 compared with PBS, ^#^*P* < 0.05 compared with rmCIRP 1 hours, and ^†^*P* < 0.05 compared with rmCIRP 2 hours. (**B**) RAW264.7 cells (8 × 10^5^ cells/mL) were stimulated with 2 and 4 μg/mL rmCIRP for 5 hours. Cells were harvested for protein extraction, followed by Western blot assays using Abs against p-STAT3, STAT3, and β–actin. Data are expressed as mean ± SEM (*n* = 3–5 wells/group). Experiments were repeated, and the repeated experimental data are shown in Supplemental Figure 8. *P < 0.05 compared with PBS, and ^#^*P* < 0.05 compared with rmCIRP (2 μg/mL). (**C**) Splenocytes isolated from healthy mice (2 × 10^6^ cells/mL) were stimulated with rmCIRP (4 μg/mL) for 10, 24, and 48 hours. Cells were harvested for protein extraction, followed by Western blot using Abs against p-STAT3, STAT3, and β-actin. Data are expressed as mean ± SEM (*n* = 3 samples/group). Experiments were repeated, and the repeated experimental data are shown in Supplemental Figure 8. *P < 0.05 compared with PBS; ^#^*P* < 0.05 compared with rmCIRP 10 hours. (**D**) Mice were injected with normal saline or rmCIRP (5 mg/kg BW) i.p. After 24 hours of PBS or rmCIRP injection, peritoneal macrophages were isolated for total protein extraction. Western blot was performed to determine p-STAT3, STAT3, and β-actin levels in each sample. Data are expressed as mean ± SEM (*n* = 3 mice/group). Experiments were repeated, and the repeated experimental data are shown in Supplemental Figure 8. *P < 0.05 compared with saline injection. Representative Western blots for p-STAT3, STAT3, and β-actin are shown. p-STAT3 expression in each sample was normalized to total STAT3 expression, and the mean values of PBS-treated groups were standardized as one for comparison. Data are expressed as mean ± SEM (*n* = 3 samples/group). The groups were compared by 1-way ANOVA and SNK method in multiple-group comparisons. Two groups were compared by 2-tailed Student’s *t* test.

STAT3 has been shown to play an important role in macrophage immune tolerance (17). In order to explore the direct effect of eCIRP on STAT3, we assessed STAT3 activation/phosphorylation in RAW264.7 macrophages after treatment with rmCIRP. We found that the amount of phosphorylated STAT3 (p-STAT3) was significantly increased in RAW264.7 macrophages after treatment with rmCIRP in a time- and dose-dependent manner compared with PBS-treated macrophages (Figure 2, A and B). Similarly, in splenocytes isolated from a normal healthy mouse, treatment with rmCIRP significantly increased the activation of STAT3 in a time-dependent manner (Figure 2C). Because RAW264.7 cells robustly increase their inflammatory markers upon treatment with TLR agonists, we chose to assess STAT3 activity at a comparatively earlier time point (≤5 hours) after rmCIRP stimulation than in primary mouse splenocytes (Figure 2, A–C). However, we also observed an increase of p-STAT3 in splenocytes after treatment with rmCIRP for 5 hours (Supplemental Figure 1; supplemental material available online with this article; https://doi.org/10.1172/jci.insight.133715DS1). We further injected mice with rmCIRP i.p. and assessed STAT3 activation in the peritoneal macrophages 24 hours later. There was increased activation of STAT3 in the macrophages isolated from rmCIRP-treated mice as compared with saline-treated mice (Figure 2D). Collectively, these data demonstrate that eCIRP serves as a novel inducer of STAT3 activation in macrophages.

### Inhibiting STAT3 reverses eCIRP-induced macrophage endotoxin tolerance.

**Figure 3:**
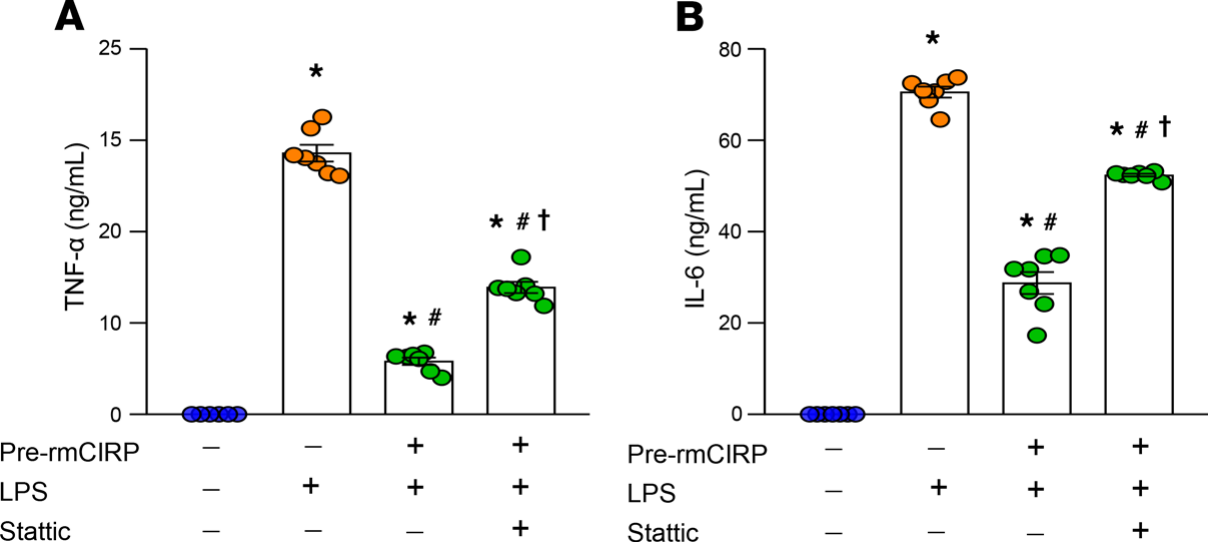
Inhibition of STAT3 by Stattic rescues macrophages from eCIRP-induced endotoxin tolerance. RAW264.7 cells (5 × 10^5^/mL) were pretreated with PBS or rmCIRP (1 μg/mL) in the absence or presence of Stattic (3 μM) for 24 hours. Cells were then washed with medium to remove rmCIRP in the supernatants, and they were further stimulated with LPS (10 ng/mL). After 5 hours, culture supernatants were collected and assessed for (**A**) TNF-α and (**B**) IL-6. Data are expressed as mean ± SEM (*n* = 7 samples/group). The experiments were performed 3 times, and all data were used for analysis. The groups were compared by 1-way ANOVA and SNK method. *P < 0.05 vs. pre-rmCIRP (–), LPS (–); ^#^*P* < 0.05 vs. pre-rmCIRP (–), LPS (+); ^†^*P* < 0.05 vs. pre-rmCIRP (+), LPS (+).

Stattic, a selective inhibitor of STAT3, prevents activation, dimerization, and nuclear translocation of STAT3 by interacting with the SH2 domain. We found that pretreatment of RAW264.7 cells with rmCIRP induced endotoxin tolerance. This was evidenced by significantly decreased levels of TNF-α and IL-6, 80% and 58%, respectively, in the supernatants of pretreated LPS-stimulated cells as compared with untreated LPS-stimulated controls (Figure 3, A and B). Treating macrophages with rmCIRP and Stattic mitigated the development of endotoxin tolerance (Figure 3, A and B). Thus, eCIRP-induced macrophage endotoxin tolerance can be partially corrected by the inhibition of STAT3.

### Discovery of IL-6R as a potentially novel biologically active receptor of eCIRP.

**Figure 4:**
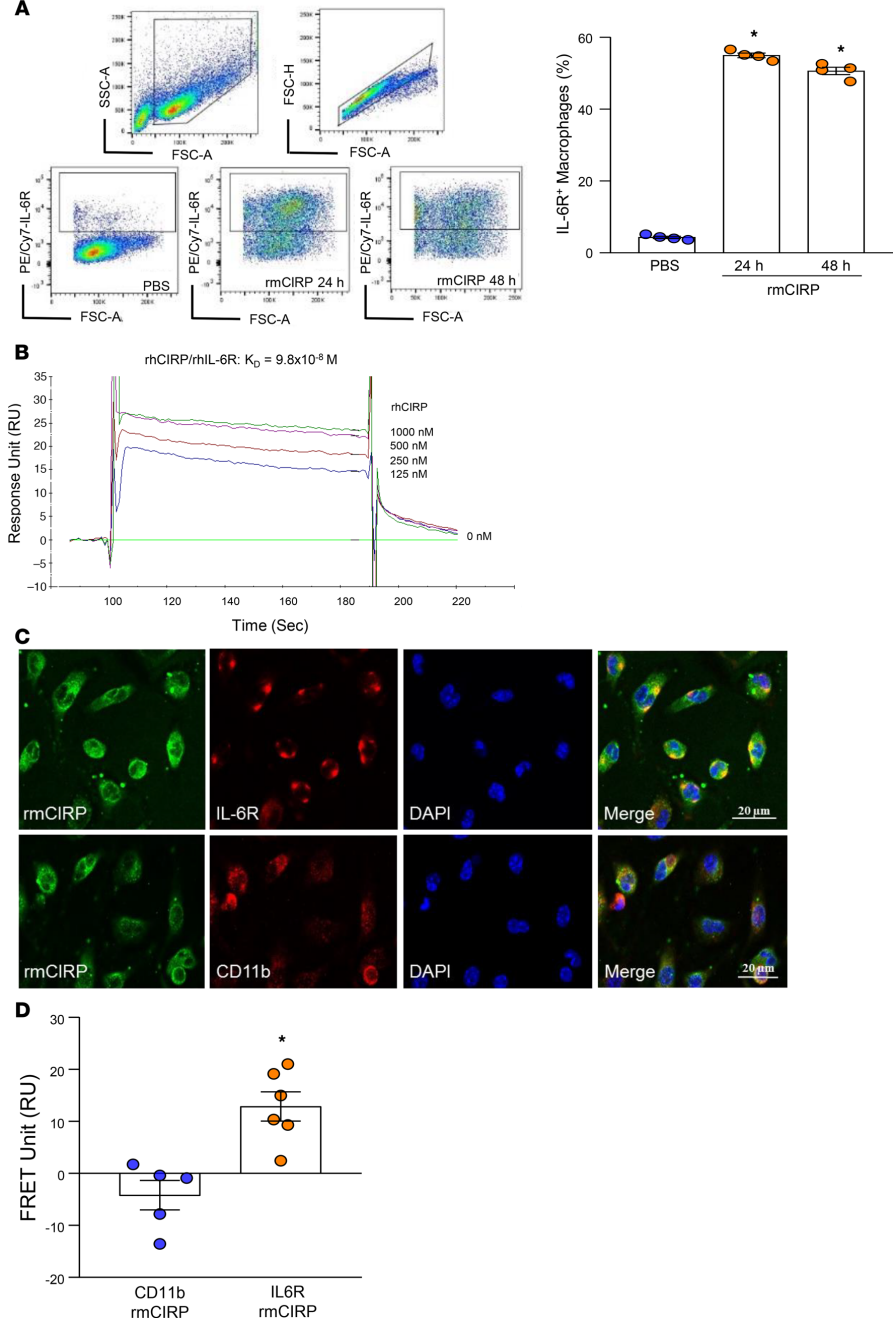
Identification of IL-6R as a potentially novel receptor of eCIRP in macrophages. (**A**) RAW264.7 cells (1 × 10^6^/mL) were stimulated with rmCIRP (1 μg/mL), and surface expression of IL-6R was assessed at 24 and 48 hours following rmCIRP treatment by flow cytometry. Data are expressed as mean ± SEM (*n* = 4 samples/group). Experiments were repeated, and the repeated experimental data are shown in Supplemental Figure 9. The groups were compared by 1-way ANOVA and SNK method (*P < 0.05 vs. PBS). (**B**) SPR (Biacore assay) was performed between rhCIRP and rhIL-6R. rhIL-6R as a ligand was immobilized on the chip. rhCIRP was injected as an analyte in concentrations of 0–1000 nM. The association and dissociation of analyte with ligand at the indicated concentration were recorded, and binding kinetics of rhCIRP and rhIL-6R was calculated. (**C**) Peritoneal macrophages (4 × 10^5^/mL) were treated with rmCIPR (5 μg/mL) at 4°C for 10 minutes, immediately fixed with paraformaldehyde, and stained with rabbit anti-mouse CIRP Ab, goat anti–mouse IL-6R Ab, and goat anti–mouse CD11b Ab followed by Cy3-conjugated donkey anti–rabbit IgG and Cy5-conjugated donkey anti–goat IgG. The images were obtained by using a Zeiss confocal microscope under 63× objective. The colocalization of rmCIRP and IL-6R is indicated by the merged images (shown in yellow color). Scale bar: 20 μm. (**D**) After the staining protocol described in (**C**), cell-associated fluorescence was measured on a Biotek Synergy Neo2 at 579 nm upon excitation at 540 nm (*E1*), at 681 nm after excitation at 640 nm (*E2*), and at 681 nm after excitation at 540 nm (*E3*) for FRET unit calculation. The transfer of fluorescence was calculated as FRET units. FRET unit = (*E*3_both_ − *E*3_none_) − ([*E*3_Cy5_ − *E*3_none_] × [*E*2_both_/*E*2_Cy5_]) − ([*E*3_Cy3_ − *E*3_none_] × [*E*1_both_/*E*1_Cy3_]). Data are expressed as mean ± SEM (*n* = 5–6 wells/group). Experiments were repeated 3 times. Groups compared by 2-tailed Student’s *t* test (*P < 0.01 vs. CD11b).

IL-6R signaling promotes STAT3 activation (18). We therefore aimed to determine whether eCIRP has any interaction with IL-6R. We found a dramatic increase in the expression of IL-6R on the surface of RAW264.7 cells following treatment with rmCIRP at both 24 and 48 hours compared with PBS-treated cells (Figure 4A). To study the direct interaction between eCIRP and IL-6R, we performed surface plasmon resonance (SPR), also known as a Biacore assay, which demonstrated a strong binding between recombinant human CIRP (rhCIRP) and rhIL-6R, with a *K_D_* of 9.8 × 10^–8^ M (Figure 4B). The binding of eCIRP and IL-6R was even stronger than the binding between IL-6R and its putative ligand, IL-6, whose *K_D_* was shown to be higher (35) than the *K_D_* of eCIRP’s binding to IL-6R. Of note, murine and human CIRP exhibit 95% amino acid sequence homology. We next performed an immunofluorescence study to confirm the colocalization of eCIRP and IL-6R in macrophages after rmCIRP stimulation. It clearly demonstrated the colocalization of rmCIRP and IL-6R, as indicated by the merged (shown in yellow) image (Figure 4C). Conversely, rmCIRP did not colocalize with a negative control, the pan-macrophage marker CD11b (Figure 4C). We also performed fluorescence resonance energy transfer (FRET) analysis to quantitatively determine rmCIRP’s association with IL-6R. FRET analysis revealed a clear association between rmCIRP and IL-6R with an increase in FRET units of 17-fold compared with rmCIRP’s interaction with negative control CD11b (Figure 4D). These findings reveal that eCIRP is a ligand of IL-6R.

### Blocking IL-6R by its neutralizing Ab attenuates eCIRP-induced STAT3 activation and corrects macrophage endotoxin tolerance.

**Figure 5:**
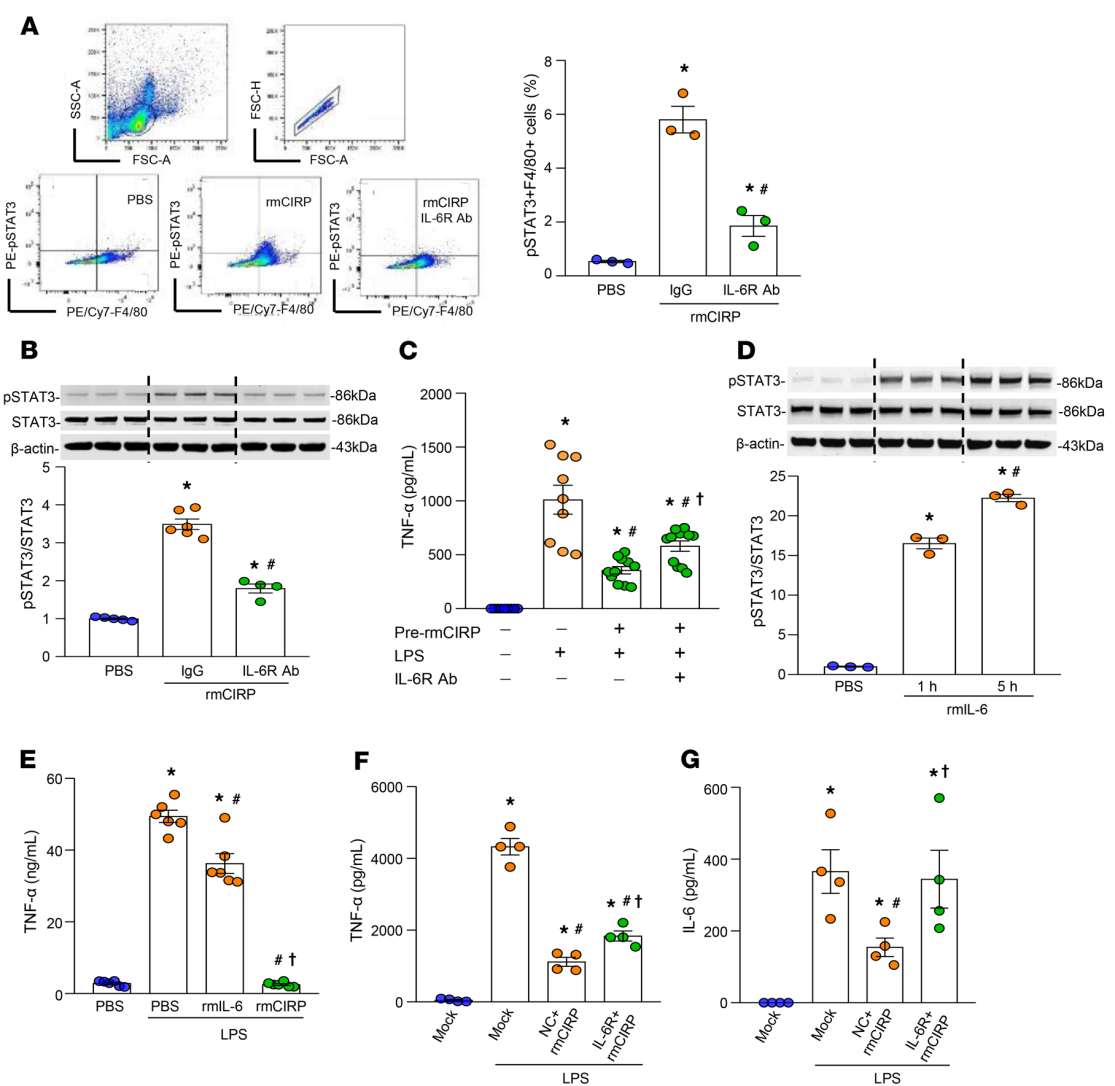
Inhibition of IL-6R corrects eCIRP-induced endotoxin tolerance. (**A**) Splenocytes were pretreated with IgG or anti–IL-6R Ab and stimulated with rmCIRP for 5 hours. Cells were fixed, permeabilized, and stained with anti–p-STAT3 and F4/80 Abs and analyzed. Data are expressed as mean ± SEM (*n* = 3 mice/group). The groups were compared by 1-way ANOVA and SNK method (*P < 0.05 vs. PBS; ^#^*P* < 0.05 vs. IgG + rmCIRP). Experiments were repeated, and the repeated experimental data are shown in Supplemental Figure 9. (**B**) Peritoneal macrophages were pretreated with IgG or anti–IL-6R Abs for 30 minutes and stimulated with PBS or rmCIRP for 24 hours. Total proteins were subjected to Western blotting using anti–p-STAT3, STAT3, and β-actin Abs. Data are expressed as mean ± SEM (*n* = 4 samples/group). The groups were compared by 1-way ANOVA and SNK method (*P < 0.05 vs. PBS; ^#^*P* < 0.05 vs. IgG + rmCIRP). Experiments were repeated, and the repeated experimental data are shown in Supplemental Figure 10. (**C**) Peritoneal macrophages were pretreated with PBS or rmCIRP with IgG or anti–IL-6R Ab for 24 hours. Cells were washed with medium and restimulated with LPS for 5 hours. TNF-α levels in the supernatants were assessed. Data are expressed as mean ± SEM (*n* = 9–11 samples/group). Results were pooled from 2 independent experiments. The groups were compared by 1-way ANOVA and SNK method. *P < 0.05 vs. pre-rmCIRP (–), LPS (–); ^#^*P* < 0.05 vs. pre-rmCIRP (–), LPS (+); ^†^*P* < 0.05 vs. pre-rmCIRP (+), LPS (+). (**D**) RAW264.7 cells were treated with rmIL-6 for 1 and 5 hours. Total protein was extracted and subjected to Western blotting using p-STAT3, STAT3, and β-actin Abs. Data are expressed as mean ± SEM (*n* = 3 samples/group). The groups were compared by 1-way ANOVA and SNK method. *P < 0.05 compared with PBS-treated cells; ^#^*P* < 0.05 compared with rmIL-6 at 1 hour. (**E**) RAW264.7 cells were treated with rmIL-6 or rmCIRP for 20 hours and were restimulated with LPS for 5 hours, and TNF-α levels in the medium were assessed. Data are expressed as mean ± SEM (*n* = 6 samples/group). The groups were compared by 1-way ANOVA and SNK method. *P < 0.05 vs. PBS (+), LPS (–); ^#^*P* < 0.05 vs. PBS (+), LPS (+); ^†^*P*<0.05 vs. LPS (+), rmIL-6 (+). (**F** and **G**) RAW264.7 cells were transfected with mock, IL-6R siRNA, or negative control (NC) siRNA and treated with rmCIRP for 20 hours. Cells were restimulated with LPS for 5 hours and (**F**) TNF-α and (**G**) IL-6 levels in the culture medium were assessed. Data are expressed as mean ± SEM (*n* = 4 samples/group). Experiments were performed twice, and all data were used for analysis. The groups were compared by 1-way ANOVA and SNK method. *P < 0.05 vs. mock (+), LPS (–); ^#^*P* < 0.05 vs. mock (+), rmCIRP (–), LPS (+); ^†^*P* < 0.05 vs. NC (+), rmCIRP (+), LPS (+).

After we confirmed eCIRP is a ligand of IL-6R (Figure 4), we aimed to further study the biological significance of the eCIRP–IL-6R interaction on STAT3 activation and macrophage endotoxin tolerance. To accomplish this, we pretreated primary murine splenocytes with anti–IL-6R neutralizing Ab or isotype IgG and, after stimulating these cells with rmCIRP, we assessed intracellular STAT3 activity. We found that splenocytes pretreated with anti–IL-6R Ab showed significant decreases in the frequencies (%) of p-STAT3^+^ macrophages (F4/80^+^) by 68% compared with isotype IgG–treated splenocytes after rmCIRP stimulation (Figure 5A). Similarly, the murine primary peritoneal macrophages pretreated with anti–IL-6R Ab showed significant decreases in the levels of p-STAT3 compared with isotype IgG–treated macrophages after stimulation with rmCIRP for 24 hours (Figure 5B). Interestingly, although TLR4 serves as one of the receptors of eCIRP, the effects of rmCIRP on STAT3 activation in wild-type (WT) macrophages treated with anti-TLR4 neutralizing Ab or in TLR4^–/–^ macrophages treated with IL-6R Ab did not show a significant difference compared to either isotype IgG–treated or WT macrophages (Supplemental Figure 2, A and B). Furthermore, immunostaining studies showed that the binding of rmCIRP to IL-6Rα was not impaired in TLR4^–/–^ macrophages (Supplemental Figure 2C). Finally, we studied the effect of anti–IL-6R Ab on eCIRP-induced macrophage endotoxin tolerance. We found that the peritoneal macrophages with anti–IL-6R Ab treatment partially corrected the endotoxin tolerance by 63% compared with the peritoneal macrophages with the isotype IgG treatment (Figure 5C). These results suggest that blocking IL-6R inhibits eCIRP-induced STAT3 activation and reverses endotoxin tolerance.

We further confirmed the critical role of IL-6R on macrophage endotoxin tolerance by using positive and negative controls. We treated macrophages with rmIL-6, which served as a positive control, and with siRNA of murine IL-6R to inhibit IL-6R expression to serve as a negative control. We found that treating macrophages with rmIL-6 significantly increased p-STAT3 in a time-dependent manner compared with PBS-treated cells (Figure 5D). We also found that the macrophages pretreated with rmIL-6 demonstrated endotoxin tolerance (Figure 5E). Interestingly, we noticed more potent LPS tolerance in rmCIRP-pretreated macrophages as compared with rmIL-6–pretreated macrophages (Figure 5E), which could be due to the possible interaction of eCIRP with its other receptor(s), such as TLR4 (27). We transfected macrophages with siRNA for IL-6R, which resulted in significant inhibition of the expression of IL-6R at protein levels (Supplemental Figure 3). Macrophages with decreased expression of IL-6R failed to exhibit substantial endotoxin tolerance, while the macrophages treated with negative control siRNA still demonstrated endotoxin tolerance after pretreatment with rmCIRP (Figure 5, F and G). These data clearly demonstrate the pivotal role of the eCIRP–IL-6R axis in macrophage endotoxin tolerance.

### eCIRP polarizes macrophages toward an M2 phenotype through IL-6R.

**Figure 6:**
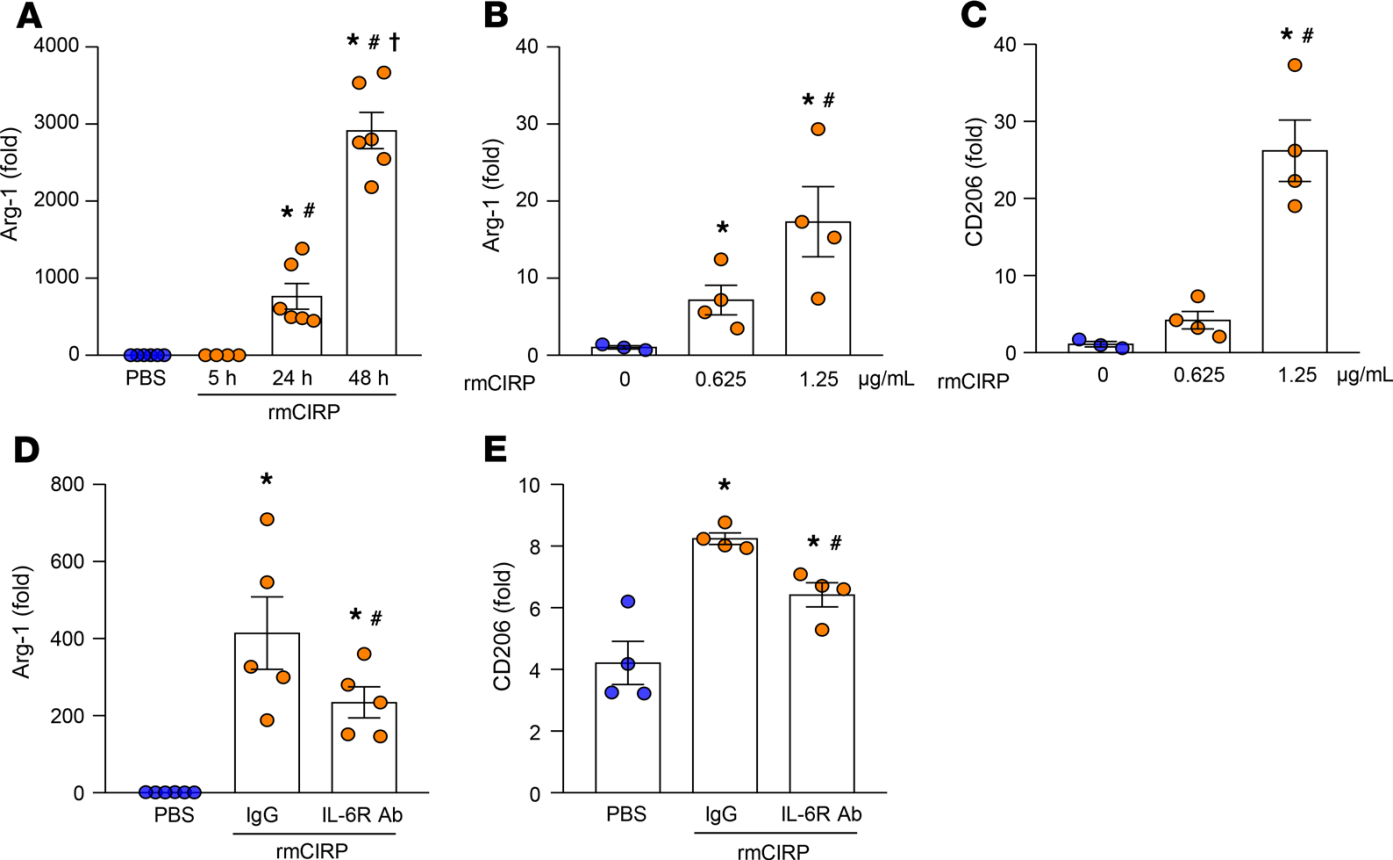
eCIRP induces M2 polarization through IL-6R. (**A**) RAW264.7 cells (1 × 10^6^/mL) were treated with rmCIRP (1 μg/mL) for 5, 24, or 48 hours. Arg-1 expression at the mRNA level was assessed by quantitative (qPCR). Expression of Arg-1 was normalized to β-actin expression and represented as fold induction compared with the normalized values of PBS control–treated cells. Data are expressed as mean ± SEM (*n* = 4–6 samples/group). Experiments were repeated, and the repeated experimental data are shown in Supplemental Figure 11. The groups were compared by 1-way ANOVA and SNK method. *P < 0.05 vs. PBS (control); ^#^*P* < 0.05 vs. rmCIRP (5 hours); ^†^*P* < 0.05 vs. rmCIRP (24 hours). (**B** and **C**) RAW264.7 cells (1 × 10^6^/mL) were treated with rmCIRP at doses of 0.625 and 1.25 μg/mL for 48 hours; the expression of Arg-1 and CD206 mRNAs was assessed by qPCR and normalized to β-actin expression. Results are represented as fold induction compared with the normalized values of PBS control–treated cells. Data are expressed as mean ± SEM (*n* = 4 samples/group). Experiments were repeated, and the repeated experimental data are shown in Supplemental Figure 11. The groups were compared by 1-way ANOVA and SNK method. *P < 0.05 vs. rmCIRP (0 μg/mL or PBS); ^#^*P* < 0.05 vs. rmCIRP (0.625 μg/mL). (**D** and **E**) RAW264.7 cells (1 × 10^6^/mL) were pretreated with IgG (3 μg/mL) or anti–IL-6R Ab (3 μg/mL) for 30 minutes. These cells were then stimulated with PBS or rmCIRP (1 μg/mL) for 24 hours, and then Arg-1 and CD206 were assessed by qPCR and flow cytometry, respectively. Arg-1 mRNA was normalized to β–actin, and data expressed in fold induction were compared with the PBS-treated condition. Data are expressed as mean ± SEM (*n* = 4–6 samples/group). Experiments were repeated 2 times, and all data were used for analysis. The groups were compared by 1-way ANOVA and SNK method (*P < 0.05 vs. PBS; ^#^*P* < 0.05 vs. IgG + rmCIRP).

**Figure 7:**
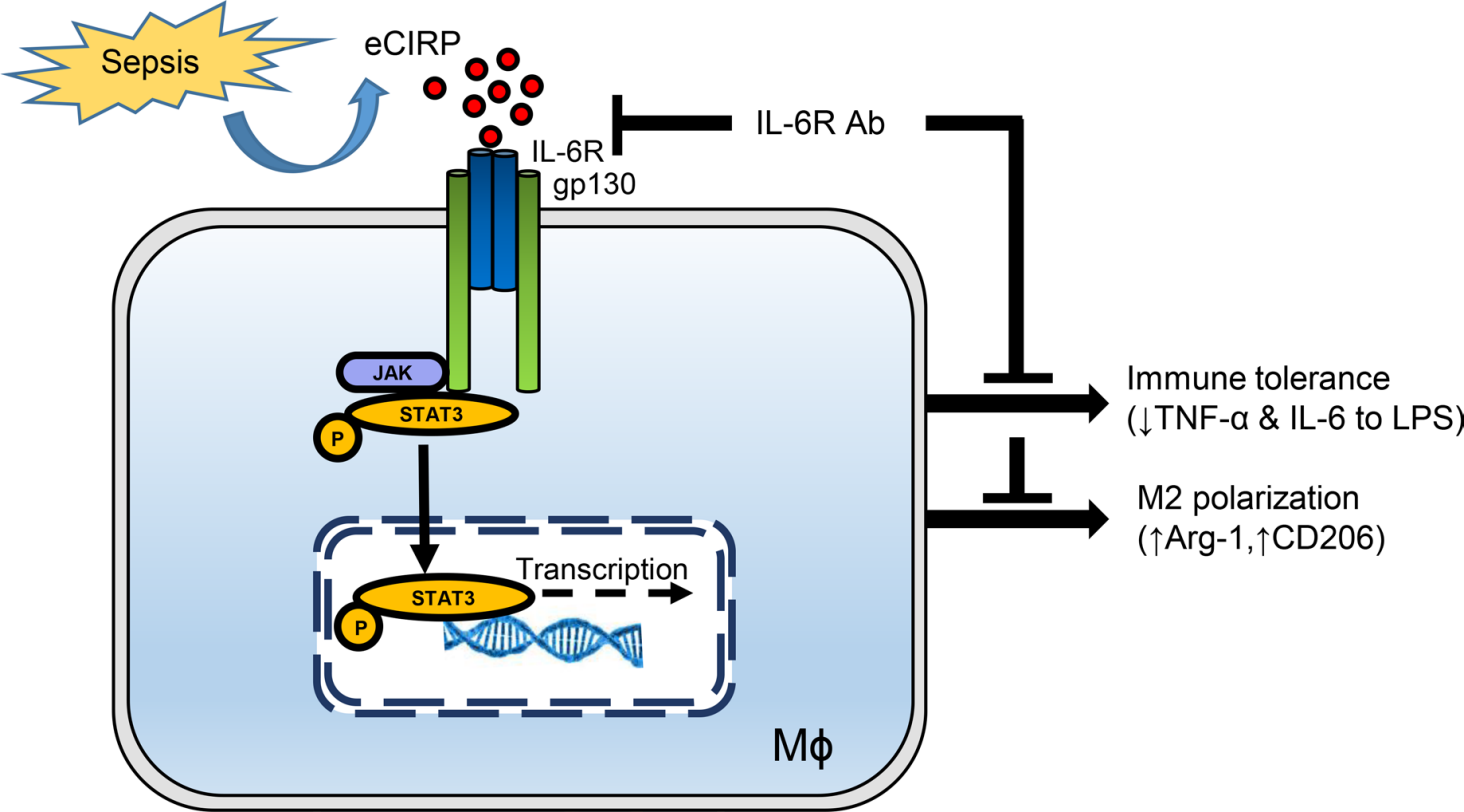
Hypothesis schema. eCIRP promotes macrophage endotoxin tolerance. eCIRP is increased during sepsis or other disease conditions and recognizes its novel receptor, IL-6R, expressed in macrophages. This leads to the activation of downstream transcription factor STAT3, which results in immune tolerance as depicted by decreased levels of TNF-α and IL-6 following LPS stimulation to these macrophages. eCIRP treatment of macrophages also induces regulatory phenotype M2 polarization in macrophages through IL-6R–dependent STAT3 activation. Inhibition of IL-6R by using its neutralizing Ab decreases eCIRP-induced STAT3 activation in macrophages and corrects immune tolerance and M2 polarization.

Because the characteristic features of M2 macrophages resemble those of endotoxin-tolerant macrophages, we assessed the M2 markers in rmCIRP-treated RAW264.7 cells at various time points (5, 24, and 48 hours) of rmCIRP treatment. We found that the expression of arginase-1 (Arg-1) mRNA was dramatically increased at the later time points (24 and 48 hours) of rmCIRP stimulation compared with PBS-treated cells (Figure 6A). We also found that treatment of RAW264.7 cells with rmCIRP increased the expression of M2 markers Arg-1 and CD206 in a dose-dependent manner (Figure 6, B and C). Morphological change serves as one of the markers of macrophage polarization (36). The morphology of RAW264.7 cells was changed to an M2 phenotype, as demonstrated by their larger size than M1 macrophages (Supplemental Figure 4A). These data were consistent with the finding of significantly increased frequencies (%) of CD206 in the peritoneal macrophages isolated from in vivo rmCIRP-injected (i.p.) mice (Supplemental Figure 4B). Conversely, if the IL-6R in RAW264.7 cells was blocked with anti–IL-6R Ab treatment, the expression of Arg-1 mRNA and the frequencies (%) of CD206 were significantly decreased by 43% and 22%, respectively, compared with isotype IgG–treated cells following stimulation with rmCIRP (Figure 6, D and E). Taken together, eCIRP released during inflammation recognizes its novel receptor IL-6R to activate STAT3, which leads to macrophage endotoxin tolerance and macrophage M2 polarization. Blocking IL-6R with neutralizing Ab abrogates these phenomena in macrophages (Figure 7).

## Discussion

In sepsis, the overwhelming inflammatory response is accompanied by the subsequent development of immune tolerance, which results in additional detrimental outcomes (32). In the current study, we discovered a new receptor of eCIRP, IL-6R, which played a critical role in macrophage tolerance. We found a dramatic increase in the expression of IL-6R in rmCIRP-treated macrophages. On the other hand, the macrophage expression of IL-10R, which also plays a pivotal role in immune regulation, was markedly lower than the expression of IL-6R in both basal and rmCIRP-treated conditions (Supplemental Figure 5). We further verified the biological function of the eCIRP–IL-6R interaction via STAT3 activation and the development of macrophage endotoxin tolerance. Interestingly, we noticed that blockade of IL-6R with its neutralizing Ab dramatically reduced p-STAT3 in rmCIRP-treated macrophages and improved immune responsiveness following LPS stimulation. In addition, we found that the inhibition of IL-6R prevented eCIRP-induced macrophage M2 polarization, thus strongly implicating the IL-6R–STAT3 axis for eCIRP-mediated immune regulation. In the present study, although we did not focus on how eCIRP increased the expression of IL-6R, we speculate that increased expression of IL-6R in macrophages after eCIRP stimulation could be through TLR4, as well as possibly by a positive feedback loop after binding to IL-6R.

The scientific premise of this study initiated with our finding that septic mice with higher serum levels of eCIRP also demonstrated immune tolerance because they contained splenocytes that produced decreased levels of the inflammatory cytokine TNF-α after ex vivo treatment with endotoxin. We have shown direct evidence of eCIRP-mediated macrophage endotoxin tolerance in primary macrophages, as well as in the RAW264.7 cell line. In both cell populations, we observed decreased levels of TNF-α and IL-6 after treatment with LPS in cells that had been pretreated with rmCIRP. These findings were consistent with the in vivo results; peritoneal macrophages from rmCIRP-injected mice produced decreased levels of IL-6 after treatment with LPS ex vivo.

In addition to endotoxin tolerance, polarization of macrophages to the M2 phenotype may contribute to immune modulation (37). It has previously been shown that the M1 to M2 macrophage reprogramming that develops during LPS tolerance resembles the pathological antiinflammatory response to sepsis (38). Here, we confirmed that the macrophages treated with rmCIRP rapidly increased the expression of the M2 markers Arg-1 and CD206, indicating these cells were skewed toward an M2 phenotype.

Our next focus was to identify a mechanism by which eCIRP induced endotoxin tolerance in macrophages. Previously, it has been shown that STAT3 plays a pivotal role in suppressing various TLR-mediated signal transduction in phagocytes (39, 40). Macrophages, neutrophils, and dendritic cells that are deficient in STAT3 produce elevated levels of proinflammatory cytokines (TNF-α, IL-6, IL-12, and IFN-γ) through the TLR4 pathway (39, 40). IL-10, an antiinflammatory cytokine, also regulates immune response. The antiinflammatory function of IL-10 is mediated through the activation of STAT3 (41). Therefore, STAT3 was a viable candidate to study eCIRP-mediated immune tolerance in macrophages. NF-κB, MAPK, and IFN regulatory factor 3 signaling cascades mainly promote proinflammatory genes’ transcription. In contrast, STAT3 has been found to induce expression of transcriptional repressors and corepressors that inhibit NF-κB gene reporters (42), suggesting an indirect mechanism by which STAT3 restrains proinflammatory gene transcription.

Immune tolerance is a common phenomenon in cancer; inhibiting immune tolerance via immunotherapy improves outcomes in cancer therapy (43). It has been shown that STAT3-deficient macrophages exhibit a constitutively activated phenotype and are more prone to produce inflammatory mediators, such as IL-6, IL-12, RANTES, macrophage inflammatory protein 1α (MIP-1α), MIP-1β, and MIP-2, in response to LPS stimulation, suggesting STAT3 signaling as a negative regulatory pathway in these cells (44). Several studies have shown the pro-oncogenic role of CIRP. CIRP was found to be overexpressed in prostate, breast, liver, and colon cancers (45–48). Downregulation of CIRP enhanced chemosensitivity and impaired survival of prostate cancer cells (45). The interaction between CIRP and STAT3 has been implicated in liver cancer, in which the tumor-bearing WT mice had a higher level of p-STAT3 than CIRP^–/–^ mice (48). Interestingly, in our study we found dramatic upregulation of p-STAT3 in macrophages and splenocytes after treatment with rmCIRP. Splenocytes contain mixed cell populations, such as macrophages and T and B cells. We found that macrophages of the spleen were more responsive to eCIRP for the induction of STAT3 phosphorylation than other cell types (Supplemental Figure 6). We further determined the impact of STAT3 signaling on eCIRP-induced macrophage endotoxin tolerance by using an inhibitor of STAT3, Stattic, which mitigated rmCIRP-induced macrophage endotoxin tolerance. We have shown that, under in vitro conditions, blocking STAT3 by Stattic partially corrected immune tolerance in eCIRP-induced RAW264.7 cells. The degree of rescue likely depends on factors such as optimal time points, which we may not have captured. Several factors that influence RAW264.7 cells’ activity, including confluence, passage number, and cell numbers, might influence the optimum conditions for blocking STAT3 to reverse eCIRP-induced immune tolerance. In addition, aside from STAT3-mediated tolerance, several other pathways and molecules, including TLR4-MyD88’s negative regulators IRAK-M, ST2, SOCS1, and SOCS3, are involved in tolerance induction in macrophages. eCIRP has been previously shown to recognize the TLR4-MD2 complex (27). Therefore, future studies on the involvement of these molecules will provide additional insight into the mechanism of eCIRP-mediated induction of immune tolerance in macrophages.

We next sought to determine how eCIRP induces STAT3 activation in macrophages. STAT3 is upregulated via the JAK family of proteins associated with putative receptors, such as cytokine receptors, G protein–coupled receptors, growth factor receptor, and tyrosine kinase receptors, which are recognized by a large number of cytokines (IL-6, IL-10, IFNs) and growth factors (EGF, G-CSF, GM-CSF, VEGF) (49). Here, we identified IL-6R to serve as a potentially novel receptor of eCIRP to activate downstream mediator STAT3, which in turn led to macrophage endotoxin tolerance.

Patients who survive the acute stage of sepsis often develop a chronic critical illness associated with immunosuppression, leading to high morbidity and mortality (50). In humans, increased levels of eCIRP in the serum have been shown to correlate with sepsis severity (27, 28). We discovered a link between eCIRP and IL-6R in murine macrophages to promote eCIRP-induced macrophage endotoxin tolerance; this provides a strong premise for studying eCIRP’s role in immune tolerance in patients with sepsis. We showed that anti–IL-6R Ab reversed eCIRP-induced macrophage endotoxin tolerance. Because strategies with neutralizing Ab targeting a signaling pathway might exhibit off-target effects, future discoveries of a small peptide targeting the eCIRP–IL-6R interaction could be helpful to counter eCIRP-induced immune tolerance in macrophages to safeguard patients from secondary infection. We used in vitro, in vivo, and ex vivo approaches to study eCIRP’s role in macrophage endotoxin tolerance, which opened up a new direction to validate our findings in various preclinical models susceptible to the development of immune tolerance resulting in secondary infections.

## Methods

### Reagents and Abs.

Reagents and culture mediums for cell cultures were purchased from MilliporeSigma and Thermo Fisher Scientific. Goat anti-mouse IL-6R polyclonal neutralizing Ab (catalog AF1830) and normal goat IgG (catalog AB-108-C) were purchased from R&D Systems, Bio-Techne. Anti-mouse TLR4/MD2 neutralizing Ab (clone MTS510, catalog 117608) was from BioLegend. STAT3 inhibitor (Stattic) was purchased from Santa Cruz Biotechnology and rmIL-6 was from R&D Systems, Bio-Techne. Abs for flow cytometry were PE anti-mouse p-STAT3 (Tyr705, clone 13A3-1, catalog 651004), PE/Cy7, Pacific blue anti-mouse F4/80 (clone BM8, catalog 123114 and 123124), and PE/Cy7 anti-mouse IL-6R (clone D7715A7, catalog 115814) from BioLegend. Abs for Western blotting included anti-mouse p-STAT3 (Tyr705, catalog 9131) and total STAT3 (catalog 9139) from Cell Signaling Technologies. β-actin Ab (clone AC-15, catalog A5441) from MilliporeSigma. Infrared dye–labeled secondary Abs were from Li-Cor Biosciences. For immunocytochemistry staining and FRET analysis, rabbit anti-mouse CIRP Ab (catalog 10209-2-AP) from ProteinTech was used. Goat anti-mouse CD11b Ab (catalog MBS420973) was from MyBiosource. Fluorescence-labeled secondary Ab Cy3-conjugated donkey anti–rabbit IgG (catalog 711-166-152) and Cy5-conjugated donkey anti–goat IgG (catalog 705-175-147) were from Jackson ImmunoResearch Laboratories.

### Experimental animals and sepsis induction.

Male C57BL/6 mice (9–12 weeks old) were purchased from Charles River Laboratories. Animals were housed in a temperature-controlled room with a 12-hour light/12-hour dark cycle and fed a standard Purina rodent chow diet. Mice were allowed to acclimate to the environment for at least 5 days before being used for experiments.

Sepsis was induced in mice by CLP as described previously (51). In brief, mice were anesthetized with 2% isoflurane inhalation. The abdomen was shaved and disinfected using povidone-iodine. A 1.5-cm midline incision was made, and the cecum was exposed and ligated with 4-0 silk suture 1 cm proximal from the distal cecal tip. The cecum was punctured twice with a 22-gauge needle, and a small amount of feces were extruded. The cecum then was returned to the abdominal cavity, and the wound was closed in layers. Sham group mice underwent laparotomy only. Both sham and sepsis mice received a subcutaneous injection of the antibiotic imipenem at a dose of 0.5 mg/mouse in 500 μL of normal saline and 500 μL of normal saline as resuscitation.

Analgesics and sedatives to mitigate pain and discomfort in septic mice have direct impacts on modulating immune responses in sepsis (52). In the current study to elucidate eCIRP’s role in immune tolerance, we avoided treating the animals with analgesics and sedatives. We used only male mice because of the findings of previous studies indicating sex-specific differences in sepsis (53). It has been reported that male and female sex steroids exhibit diverse immune-modulating functions under normal conditions and varied disease processes (53). Experimental studies in mice revealed a significantly increased survival rate of female mice following polymicrobial sepsis induced by CLP compared with male animals (54). Therefore, the immuno-neuroendocrine system that varies between male and female sex may not be ignored while making a CLP model in animals to study sepsis pathogenesis.

### In vivo administration of rmCIRP.

rmCIRP was prepared in-house and the quality control assays were performed as described previously (27). The quality of the purified protein was assessed by Ponceau staining of the gel and Western blotting. Functional assay of the protein was done by assessing the TNF-α levels in the macrophages after treating them with purified rmCIRP. The level of LPS in the purified protein was measured by a limulus amebocyte lysate (LAL) assay (Cambrex). Only the purified protein lots that were endotoxin free were considered for in vitro and in vivo experiments. We performed these quality control assays for each purified protein lot. To rule out a contribution from LPS in the inflammatory response to rmCIRP, our previous study showed that incubation with polymyxin B, an LPS-binding antibiotic, did not interfere with rmCIRP-induced production of TNF-α, whereas heat treatment reduced the activity of rmCIRP in macrophages (27). rmCIRP at a dose of 5 mg/kg BW or normal saline was administered into mice by injection (i.p.). At 24 hours after rmCIRP injection, mice were anesthetized, and peritoneal lavage was collected for macrophage isolation and analyses.

### Isolation of peritoneal macrophages and splenocytes and cell culture.

Murine peritoneal macrophages and splenocytes were isolated from healthy adult mice. Mice were anesthetized with 2% isoflurane inhalation. Peritoneal cells were isolated by washing with cold Hanks’ balanced salt solution (HBSS) without Ca^2+^ and Mg^2+^, with 5% FBS. Collected peritoneal cells were washed once with cold HBSS by centrifugation at 300 *g* for 10 minutes at 4°C followed by using 0.5 mL RBC lysing buffer (BD Biosciences) for 5 minutes at room temperature to lyse RBCs. Peritoneal cells were cultured in RPMI 1640 medium supplemented with 10% heat-inactivated fetal bovine serum (FBS), 2 mM glutamine, 100 IU/mL penicillin-streptomycin, and 25 mM HEPES (complete RPMI). Peritoneal macrophages were then allowed to adhere in the culture plates for 3 hours at 37°C in 5% CO_2_. Nonadherent cells were removed by washing with culture medium. Adhered peritoneal macrophages were then detached from the plate using a cell scraper and counted. Isolated primary cells were cultured overnight prior to use.

Spleens were collected from the mice and passed through a 70-μm nylon cell strainer using the plunger end of a 5-mL syringe. The splenocyte suspension was centrifuged at 300 *g* for 5 minutes at 4°C. The cell pellet was suspended in 1 mL RBC lysing buffer (BD Biosciences) to lyse the RBCs in the suspension, followed by the washing of the cells with PBS. The cell pellets were then resuspended into complete RPMI medium and we counted the cells.

Mouse macrophage RAW264.7 cells were obtained from American Type Culture Collection (ATCC) and cultured in Dulbecco’s modified Eagle medium (DMEM) containing 10% FBS, 2 mM glutamine, and 100 IU/mL penicillin-streptomycin. The cells were cultured at 37°C in 5% CO_2_.

### ELISA.

Serum levels of eCIRP were determined by using an ELISA kit from LifeSpan Biosciences. Cytokine levels of cell culture supernatants were analyzed by ELISA using the kits of TNF-α and IL-6 from BD Biosciences, following the protocols described by the manufacturer.

### Flow cytometry.

To analyze the expression of IL-6R and CD206 on macrophages, isolated peritoneal macrophages, splenocytes, or RAW264.7 macrophages were washed with PBS with 2% FBS (FACS buffer). To exclude any nonspecific binding, we treated cells in FACS buffer with Fc receptor (anti-mouse CD16/32, clone 93; BioLegend) for 10 minutes, before staining the cells with fluorescence-labeled Abs and respective isotype control IgGs. A BD LSRFortessa flow cytometer (BD Biosciences) was used to perform the flow cytometry. For intracellular p-STAT3 staining, cells were fixed and permeabilized, followed by staining with anti–p-STAT3 Abs or isotype IgGs. At least 3 × 10^4^ cells were collected and analyzed with FlowJo software (Tree Star). Unstained and single color-stained cells were used for setting up compensation in the measurement.

### Real-time quantitative reverse transcription PCR.

Total RNA was extracted from RAW264.7 macrophages (ATCC) using TRIzol reagent (Thermo Fisher Scientific). cDNA was synthesized using MLV reverse transcriptase (Thermo Fisher Scientific). PCR reactions were carried out in 25 μL of a final volume in SYBR Green master mix (Thermo Fisher Scientific) with 0.08 μm of each forward and reverse primers (Supplemental Table 1) and cDNA. Amplification was conducted in a StepOnePlus real-time PCR machine (Thermo Fisher Scientific) and analyzed by the 2^-ΔΔCT^ method for relative quantitation normalized to mouse β-actin mRNA expression. The relative expression of mRNA was expressed as fold change in comparison with untreated control.

### Western blotting.

Cells were harvested and lysed in lysis buffer (10 mM Tris-HCl at pH 7.5, 100 mM NaCl, 1 mM EDTA, 1 mM EGTA, 1% Triton X-100) containing protease inhibitor and phosphatase inhibitor cocktail tablet (Thermo Fisher Scientific). Cell lysates were fractionated on 4%–12% Bis-Tris gels and transferred to nitrocellulose membranes. After blocking with 0.1% casein in Tris-buffered saline, the membranes were incubated in anti-mouse p-STAT3, STAT3, and β-actin Abs overnight at 4°C. The target bands were detected by using infrared dye–labeled secondary Abs and Odyssey Clx image system (Li-Cor Biosciences). The intensities of the bands were analyzed using Image Studio 5.2 software (Li-Cor Biosciences).

### Detection of the binding of eCIRP and IL-6R by SPR.

SPR technology was used to examine the interaction of eCIRP and IL-6R. SPR was conducted using a Biacore 3000 instrument (GE Healthcare) to analyze the binding between rhCIRP (Origene) and rhIL-6R (R&D Systems, Bio-Techne). According to the manufacturer, rhCIRP was produced with TrueORF clone RC201639 and was expressed in the HEK293T cell line. The protein was tagged with C-Myc/DDK and recombinant protein was captured through an anti-DDK affinity column followed by conventional chromatography steps. rhIL-6R was prepared in *Spodoptera frugiperda*, *Sf*21 baculovirus–derived human IL-6R α protein Leu20-Asp358. rhIL-6R used in the Biacore assay did not contain any tags. Endotoxin levels in the purified protein were assessed as less than 1.0 EU per 1 μg of the protein using the LAL method. The binding reaction was performed in 1× PBS buffer containing 0.01% Tween-20 (pH 7.4). The CM5 dextran chip (flow cell-2) was first activated by injection with 89 μL of 0.1 M *N*-ethyl-*N*′-[3-diethylaminopropyl]-carbodiimide and 0.1 M *N*-hydroxysuccinimide. An aliquot of 200 μL of 5 μg/mL of the ligand (rhIL-6R) diluted in 10 mM sodium acetate (pH 4.5) was injected into flow cell-2 of the CM5 chip for immobilization. Then, 135 μL of 1 M ethanolamine (pH 8.2) was injected to block the remaining active sites. The flow cell-1 without coating with the ligand was used as a control to evaluate nonspecific binding. The binding analyses were performed at a flow rate of 30 μL/min at 25°C. To evaluate the binding, the analyte rhCIRP, ranging from 0 μM (or PBS as vehicle control) to 1.0 μM for the kinetics analysis or 0.5 μM rhCIRP for the yes-or-no binding analysis, was injected into flow cell-1 and flow cell-2, and the association of analyte and ligand was recorded by SPR. The signal from the blank channel (flow cell-1) was subtracted from the channel coated with the ligand (flow cell-2). Data were analyzed by the Biacore 3000 Evaluation software. For all samples, a blank injection with buffer alone was subtracted from the resulting reaction surface data. Data were globally fitted to the Langmuir model for 1:1 binding.

### Detection of the binding of eCIRP and IL-6R by immunofluorescent staining and FRET.

Peritoneal macrophages were treated with rmCIRP (5 μg/mL) for 10 minutes at 4°C and then fixed immediately with 4% paraformaldehyde. After a brief rinse with PBS, the cells were incubated with an Ab mixture of anti-mouse CIRP (1:35) and anti-mouse IL-6R (1:30). The cells incubated in anti-mouse CIRP Ab (1:35) with anti-mouse CD11b Ab (1:50) served as a control for the colocalization of rmCIRP and IL-6R. Confocal microscopy images were obtained using a Zeiss LSM880 confocal microscope under a 63× objective (Zeiss).

The interaction of eCIRP and IL-6R was further analyzed employing FRET technology (55). Peritoneal macrophages in a 96-well plate were treated with rmCIRP for 10 minutes at 4°C and fixed with 4% paraformaldehyde. Similar to the immunostaining above, after the wash, the cells were incubated in the Ab mixture of anti-mouse CIRP with anti-mouse IL-6R or anti-mouse CIRP with anti-mouse CD11b. Cy3-labeled anti–rabbit IgG and Cy5-labeled anti–goat IgG were used as secondary Abs. When the fluorophores of a FRET donor and a FRET acceptor were in proximity with the proper orientation, FRET occurred between them shown as FRET units (55). The cell-associated fluorescence was measured on a Biotek Synergy Neo2 at 579 nm upon excitation at 540 nm (*E1*), at 681 nm after excitation at 640 nm (*E2*), and at 681 nm after excitation at 540 nm (*E3*). The transfer of fluorescence, which is the binding status of the 2 molecules, was calculated as FRET units using the formula: FRET unit = (*E*3_both_ − *E*3_none_) − ([*E*3_Cy5_ − *E*3_none_] × [*E*2_both_/*E*2_Cy5_]) − ([*E*3_Cy3_ − *E*3_none_] × [*E*1_both_/*E*1_Cy3_]) (56).

### Treatment of macrophages with rmIL-6.

RAW264.7 cells were treated with rmIL-6 (50 ng/mL) for 1 and 5 hours. Total protein was extracted from each group and subjected to Western blotting using p-STAT3, STAT3, and β-actin Abs. For the tolerance assay, RAW264.7 cells were first treated with rmIL-6 (50 ng/mL) for 20 hours. After washing the cells with Opti-MEM medium (Life Technologies), cells were restimulated with LPS (10 ng/mL) for 5 hours, and TNF-α levels in the culture medium were assessed by ELISA.

### Inhibition of IL-6Rα in macrophages by siRNA transfection.

IL-6R siRNA, a pool of 3 target-specific 19- to 25-nt siRNAs designed to abrogate IL-6Rα expression, was purchased from Santa Cruz Biotechnology (catalog sc-40065). A nontargeting 20- to 25-nt siRNA was used as a control (catalog sc-37007, Santa Cruz Biotechnology). Electroporation method was used for efficient transfection of IL-6R siRNA into RAW264.7 macrophages by using Neon Transfection System (catalog MPK5000; Life Technologies). RAW264.7 cells were cultured for 1–2 days in DMEM supplemented with 10% FBS, 2 mM glutamine, and 100 IU/mL penicillin-streptomycin until cells were 70%–90% confluent. The Neon transfection system (Life Technologies), which is paired with the Neon kit (Life Technologies), was used for transfection of IL-6R siRNA in RAW264.7 cells by following the protocol of RAW264.7 cell transfection from the manufacturer. A mixture of 1 × 10^5^ cells and 50 pmol of IL-6Rα siRNA/control siRNA was taken into a 10-μL Neon tip, and the pipette was installed into the Neon pipette station for electroporation. Electroporated cells were immediately transferred into a 24-well plate containing 500 μL/well complete DMEM without penicillin-streptomycin. The parameters for electroporation were pulse voltage: 1680 volts, pulse width: 20 μs, and pulse number: 1. After 72 hours’ culture, IL-6R expression in RAW264.7 cells was determined by Western blot analysis. In an additional group, 72 hours after siRNA transfection, RAW264.7 cells were treated with rmCIRP (1 μg/mL) for 24 hours, then stimulated with LPS (10 ng/mL) for 5 hours. The release of TNF-α and IL-6 in the culture supernatants was measured by ELISA.

### Statistics.

All data are expressed as mean ± SEM. One-way ANOVA and SNK test were performed to compare among multiple groups. All data were tested for normality. For comparison of 2 groups, we performed unpaired 2-tailed Student’s *t* tests. A *P* value less than 0.05 was considered significant.

### Study approval.

All experiments were performed in accordance with the National Institutes of Health (NIH) guidelines for the use of experimental animals and were approved by the Institutional Animal Care and Use Committee of the Feinstein Institutes for Medical Research. The number of animals in each experiment was determined by using SigmaPlot 12.5 (Systat Software, Inc.), and these predications were in line with our previous publication (51).

## Author contributions

MZ and MA did experimental design. MZ and HTY performed animal work. MZ, GM, and NLD performed in vitro experiments. MZ and NLD performed immunostaining and FRET studies. MZ, MA, and PW analyzed the data. MA and MZ prepared the figures and wrote the manuscript. PW and NLD reviewed and edited the manuscript. PW conceived the idea and supervised the project.

## Supplementary Material

Supplemental data

## Version 1 02/06/2020

In-Press Preview

## Version 2 03/12/2020

Electronic publication
