# Supplementary material for: Extracellular CIRP induces macrophage endotoxin tolerance through IL-6R–mediated STAT3 activation
Source: JCI Insight. 2020 Mar 12;5(5):e133715. doi: 10.1172/jci.insight.133715 (PMC7141386; doi:10.1172/jci.insight.133715)
Supplement: Supplemental data [file jciinsight-5-133715-s076.pdf]

Supplemental Figure 1

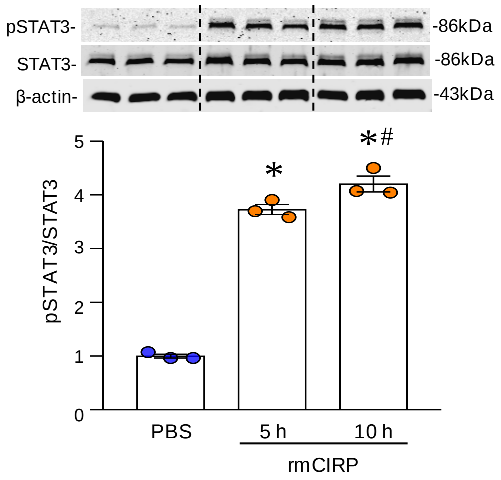

Supplemental Figure 1.

Treatment of splenocytes with rmCIRP induces pSTAT3. Splenocytes (2×10^6 cells/ml) isolated from normal healthy mice were stimulated with rmCIRP (4 μg/ml) for 5 and 10 h. Cells were harvested for protein extraction, followed by western blot using Abs against murine pSTAT3, STAT3, and β-actin. $n = 3$ independent experiments. $^*P < 0.05$ compared to PBS, $^{\#}P < 0.05$ compared to rmCIRP 5 h. Representative western blots for pSTAT3, STAT3, and β-actin are shown. Each blot was quantified by densitometric analysis. pSTAT3 expression in each sample was normalized to total STAT3 expression, and the mean values of PBS treated groups were standardized as one for comparison. All data are expressed as means ± SE. The groups were compared by one-way ANOVA and SNK method in multiple group comparisons. STAT3, signal transducer and activator of transcription 3.

Supplemental Figure 2

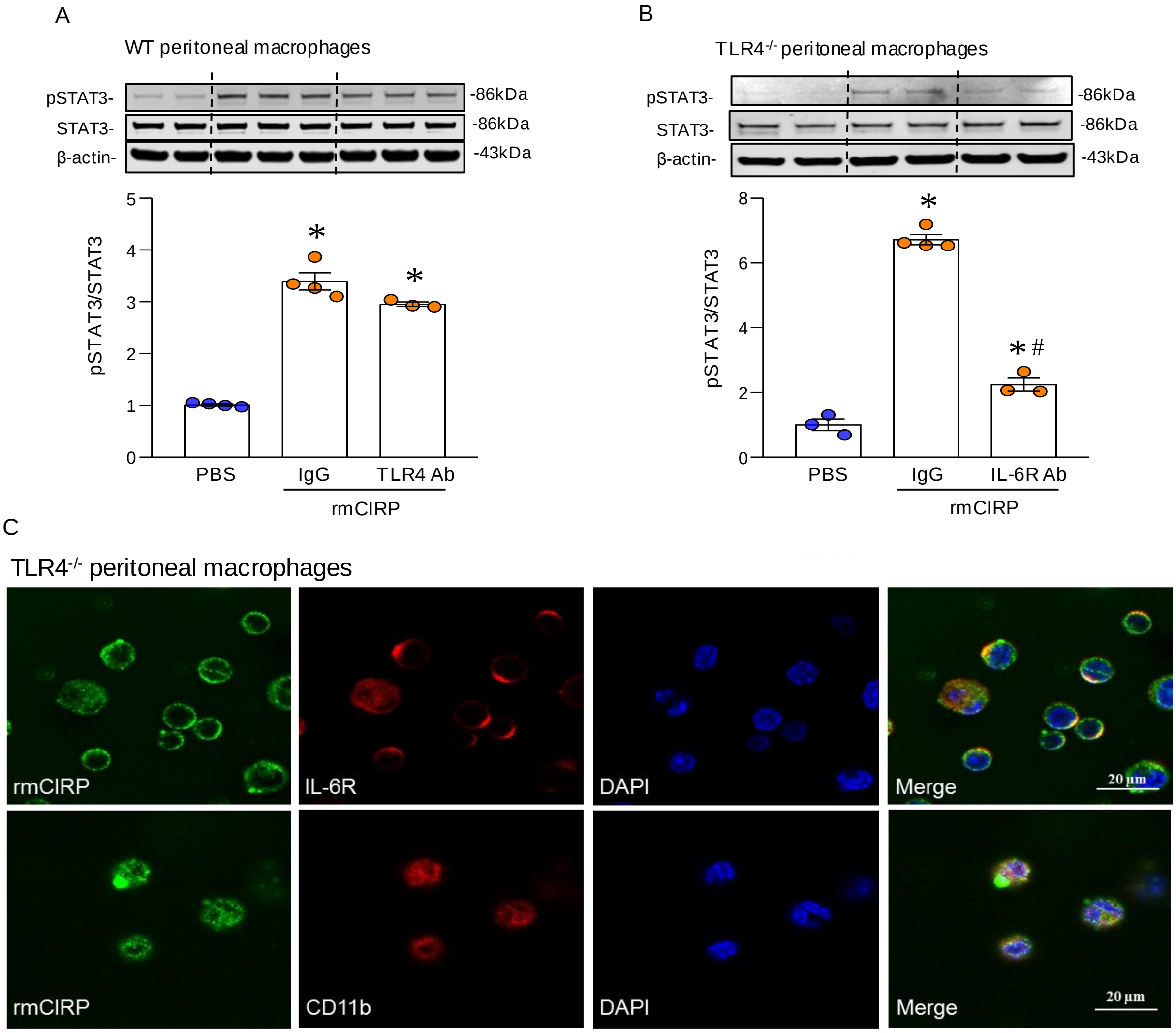

Supplemental Figure 2.

Inhibition of TLR4 does not affect STAT3 activation in rmCIRP-treated macrophages. (A) Peritoneal macrophages (1×10^6/ml) from normal healthy mice were pre-treated with IgG (3 µg/ml) or anti-TLR4 neutralizing Abs (3 µg/ml) for 30 min and then stimulated with rmCIRP (1 µg/ml) for 24 h. Total proteins were extracted from each sample and subjected to western blotting using anti-pSTAT3, STAT3, and β-actin. **(B)** Peritoneal macrophages (1×10^6/ml) isolated from TLR4$^{-/-}$ mice were pre-treated with IgG (3 µg/ml) or anti-IL-6R neutralizing Abs

(3 μg/ml) for 30 min and then stimulated with rmCIRP (1 μg/ml) for 24 h. Total proteins were extracted from each sample and subjected to western blotting using anti-pSTAT3, STAT3, and β-actin. Each blot was quantified by densitometric analysis. pSTAT3 expression in each sample was normalized to total STAT3 expression, and the mean values of PBS-treated group were standardized as one for comparison. All data are expressed as means ± SE (n=3-4 samples/group). The groups were compared by one-way ANOVA and SNK method (*$P<0.05$ vs. PBS; #$P<0.05$ vs. IgG + rmCIRP). **(C)** Peritoneal macrophages (4×10^5) from TLR4$^{-/-}$ mice were treated with rmCIRP (5 μg/ml) at 4°C for 10 min, fixed in a nonpermeabilized fashion, and stained with rabbit anti-mouse CIRP Ab, goat anti-mouse IL-6R Ab and goat anti-CD11b Ab, followed by Cy5-conjugated donkey anti-goat IgG, and Cy3-conjugated donkey anti-rabbit IgG. Confocal microscopy images were obtained at using a Zeiss LSM880 confocal microscope equipped with a 63× objective. The co-localization of rmCIRP and IL-6R indicated by the merged images (yellow color). Scale bar 20 μM. TLR4, Toll-like receptor 4; STAT3, signal transducer and activator of transcription 3.

Supplemental Figure 3

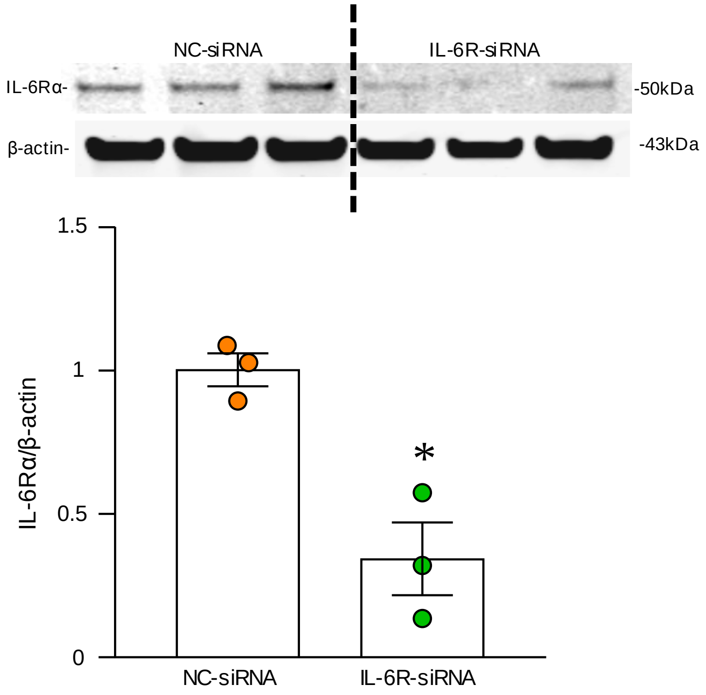

Supplemental Figure 3. Treatment with IL-6R siRNA inhibits IL-6R expression in macrophages. RAW264.7 cells were cultured for 1-2 days in DMEM supplemented with 10% FBS, 2 mM glutamine, 100 IU/ml penicillin-streptomycin until cells were 70-90% confluent. A mixture of 1×10^5 cells and 50 pmol of IL-6Rα siRNA/control siRNA was taken into a 10 µl Neon tip, and the pipette was installed into the Neon pipette station for electroporation. Electroporated cells were immediately transferred into a 24-well plate containing 500 µl/well complete DMEM medium without penicillin-streptomycin and cultured for 72 h. The cells were then stimulated with rmCIRP at a dose of 1 µg/mL for 24 h. IL-6R expression in RAW264.7 cells was determined by western blot using Abs against murine IL-6R and β-actin. Data is expressed as mean ± SE (n=3 samples/group). Experiments were repeated two times. $*P < 0.05$ compared to NC siRNA. Representative western blots for IL-6R and β-actin are shown. Each blot was quantified by densitometric analysis. IL-6R expression in each sample was

normalized to β-actin expression, and the mean values of PBS treated groups were standardized as one for comparison. All data are expressed as means ± SE. Two groups were compared by unpaired 2-tailed Student's *t* test. NC, negative control.

Supplemental Figure 4

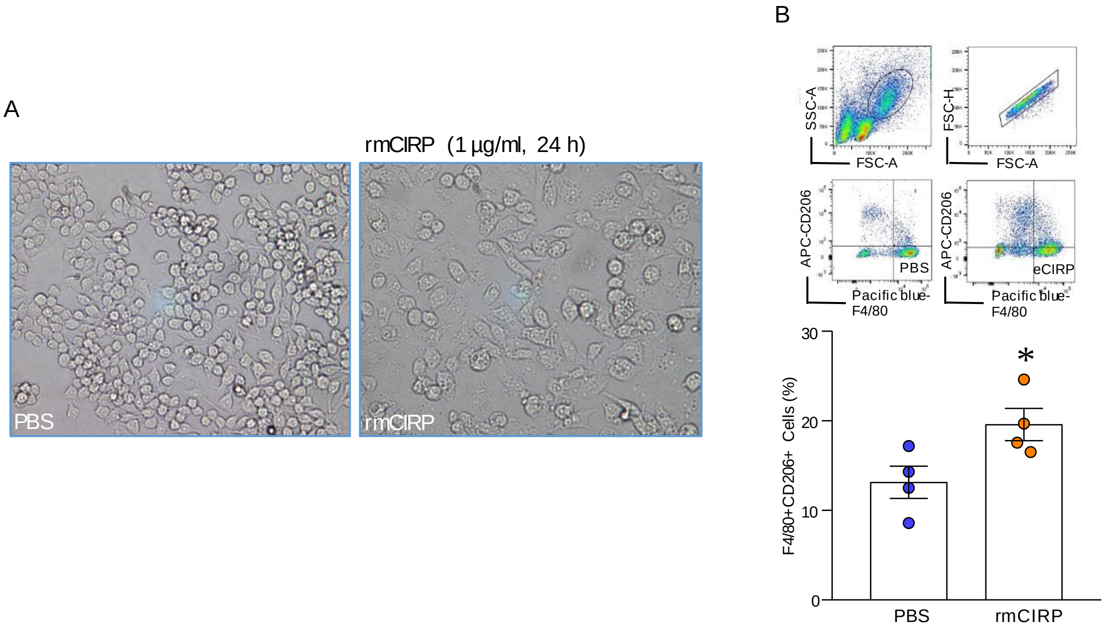

Supplemental Figure 4.

eCIRP induces CD206 expression and alters morphology in macrophages. (A) Examination of the cellular morphology of RAW264.7 macrophages (5×10^5 cells/ml) treated with rmCIRP (1 µg/ml) for 24 h by light microscopy using 40 × objective. **(B)** Mice were injected with normal saline or rmCIRP (5 mg/kg BW) intraperitoneally *(i.p.).* After 24 h of *i.p.* injection with normal saline or rmCIRP, peritoneal macrophages were isolated. A total of 1×10^6 peritoneal macrophages were surface stained with anti-F4/80 (Pacific blue conjugated) followed by permeabilization to stain CD206 (APC conjugated) Abs and assessed by flow cytometry. Data

are expressed as means ± SE (n= 4 mice/group). The groups were compared by Student's *t* test (**P*<0.05 vs. saline injection). eCIRP, extracellular cold-inducible RNA-binding protein.

Supplemental Figure 5

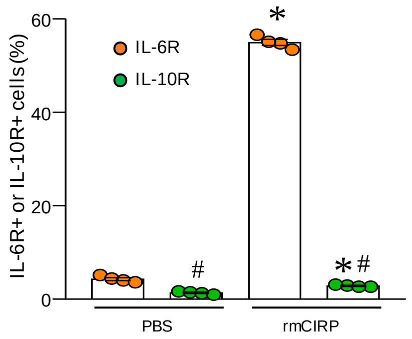

Supplemental Figure 5.

Comparison between IL-6R and IL-10R expression in rmCIRP-treated macrophages.

RAW264.7 cells (1×10^6 cells/ml) were stimulated with rmCIRP (1 µg/ml) for 24 h and then expression of IL-6R and IL-10R on the surface of macrophages were assessed by flow cytometry. Data are expressed as means ± SE (n=4 samples/group). The groups were compared by one-way ANOVA and SNK method (**P*<0.05 vs. respective PBS-treated cells; #*P*<0.05 vs. IL-6R). rmCIRP, recombinant murine cold-inducible RNA-binding protein.

Supplemental Figure 6

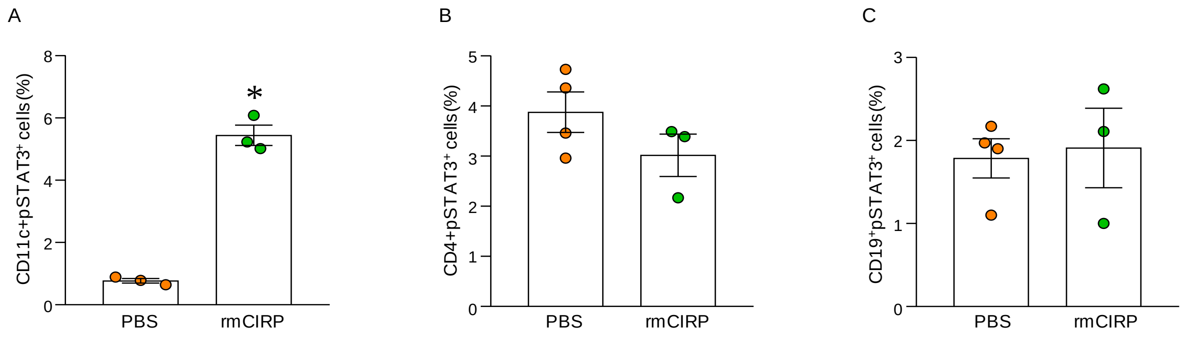

Supplemental Figure 6.

Status of eCIRP-induced STAT3 phosphorylation in various types of cells in the spleen.

Murine primary splenocytes (2×10^6/ml) were stimulated with rmCIRP (4 μg/ml) for 5 h. Cells were stained with anti-pSTAT3 Ab (PE conjugated) for intracellular pSTAT3 after fixing and permeabilizing the cells, and then surface stained with Abs for **(A)** CD11c (FITC conjugated; clone: N418, Biolegend), **(B)** CD4 (PE/Cy7 conjugated; clone: GK1.5; Biolegend), and **(C)** CD19 (pacific blue conjugated; clone: 6D5, Biolegend). The fluorescent signals from the stained cells were analyzed by flow cytometry. Data are expressed as means ± SE, n=3-4 mice/group. The groups were compared by Student *t* test (*P<0.05 vs. PBS).

Supplemental Figure 7

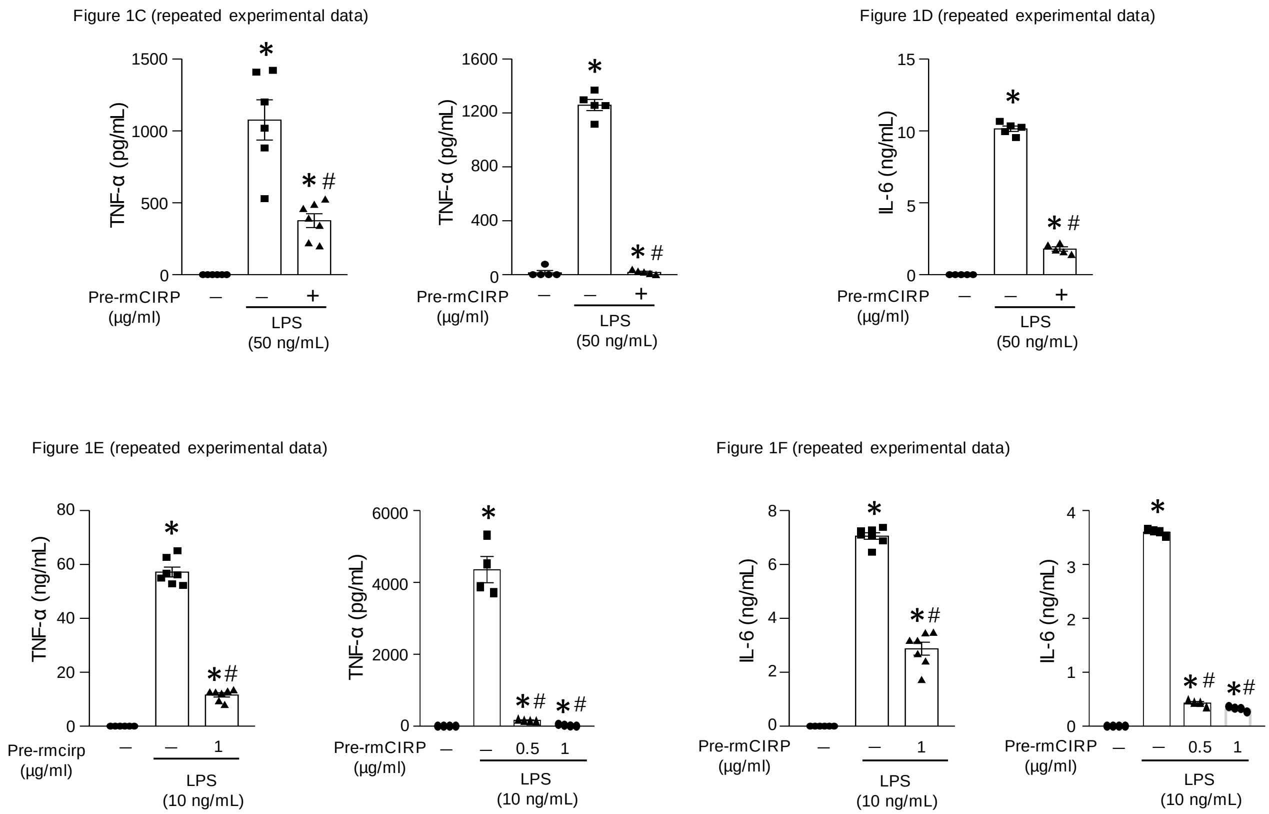

Supplemental Figure 7.

(Original Figure 1C and D repeated data) A total of 7 × 10^5/ml peritoneal macrophages isolated from healthy mice were pre-stimulated with PBS or rmCIRP (1 μg/ml) for 24 h, and the cells were washed with medium. Macrophages were re-stimulated with LPS (50 ng/ml) for 5 h and assessed for **(C)** TNF-α and **(D)** IL-6 in the culture supernatants. Data is expressed as mean ± SE. *$P < 0.05$ vs. PBS control, $^{\#}P < 0.05$ vs. pre-rmCIRP(-), LPS(+). **(Original Figure 1E, F repeated data)** RAW264.7 macrophages (3 × 10^5/ml) were pre-treated with PBS or rmCIRP at 0.5 and 1.0 μg/ml for 24 h. Cells were washed with medium, re-stimulated with LPS (10 ng/ml) for 5 h and assessed for **(E)** TNF-α and **(F)** IL-6 in the culture supernatants. Data is expressed as mean ± SE. *$P < 0.05$ vs. PBS control, $^{\#}P < 0.05$ vs. pre-rmCIRP(-), LPS(+).

Supplemental Figure 8

Figure 2A, B (repeated experimental data)

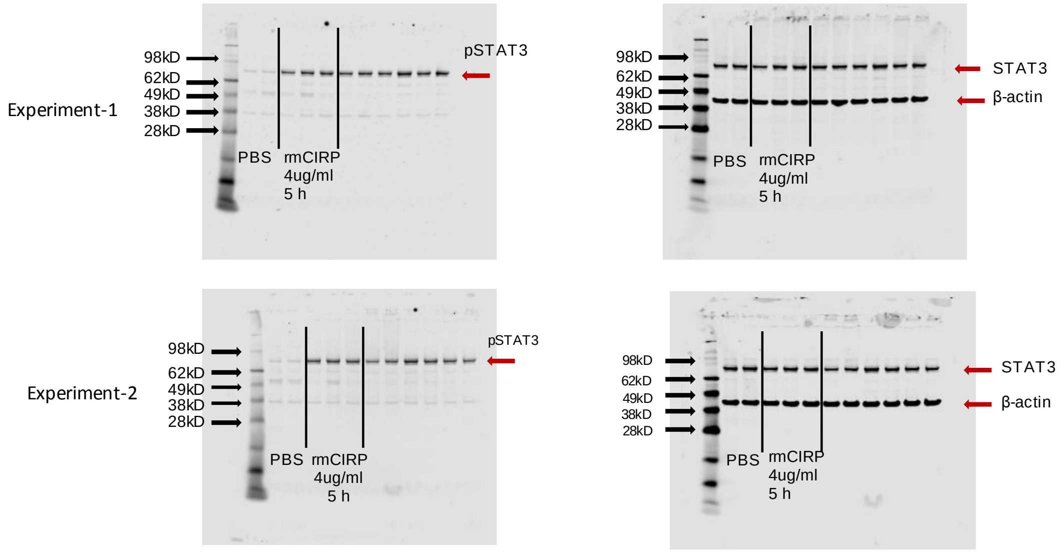

Figure 2C (repeated experimental data)

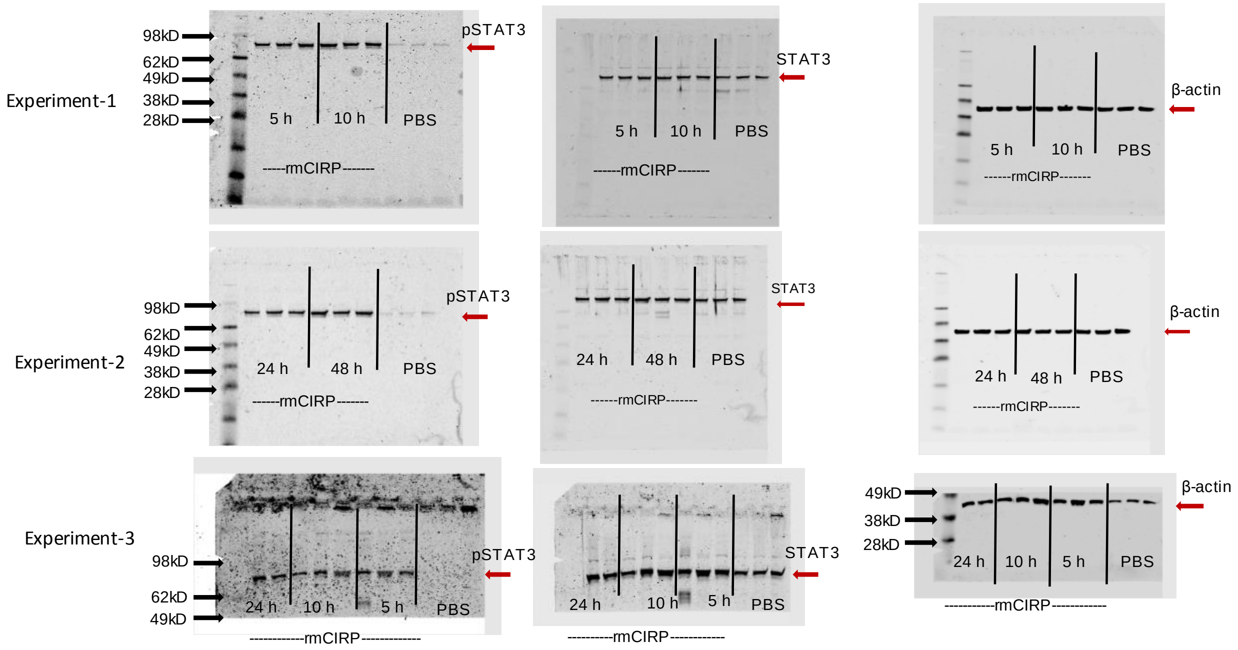

Figure 2D (repeated experimental data)

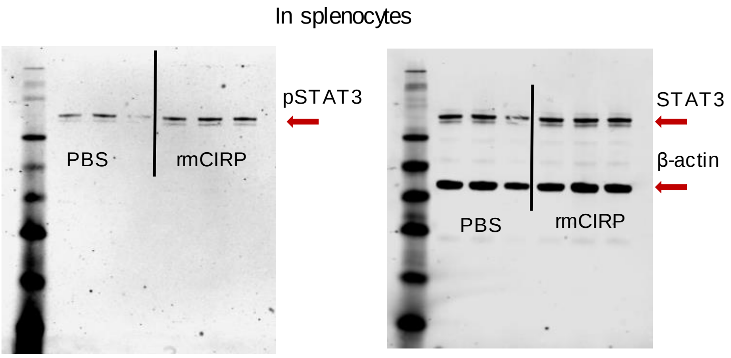

Supplemental Figure 8.

(Original Figure 2A-D repeated data) (A) RAW264.7 cells (8 × 10^5 cells/ml) were stimulated with rmCIRP (4 µg/ml) for 5 h. Cells were harvested for protein extraction, followed by western blot using Abs against pSTAT3, STAT3, and β-actin. **(B)** RAW264.7 cells (8 × 10^5 cells/ml) were stimulated with 4 µg/ml rmCIRP for 5 h. Cells were harvested for protein extraction, followed by western blot assays using Abs against pSTAT3, STAT3, and β-actin. **(C)** Splenocytes isolated from healthy mice (2 × 10^6 cells/ml) were stimulated with rmCIRP (4 µg/ml) for 5, 10, 24, and 48 h. Cells were harvested for protein extraction, followed by western blot using Abs against pSTAT3, STAT3, and β-actin. **(D)** Mice were injected with normal saline or rmCIRP (5 mg/kg BW) *i.p.*, after 24 h of PBS or rmCIRP injection, spelenocytes were isolated for total protein extraction. Western blot was performed to determine pSTAT3, STAT3, and β-actin in each sample.

Supplemental Figure 9

Figure 4A

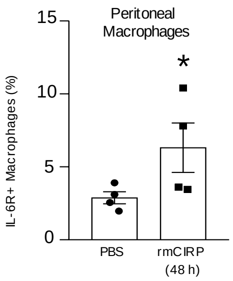

Figure 5A

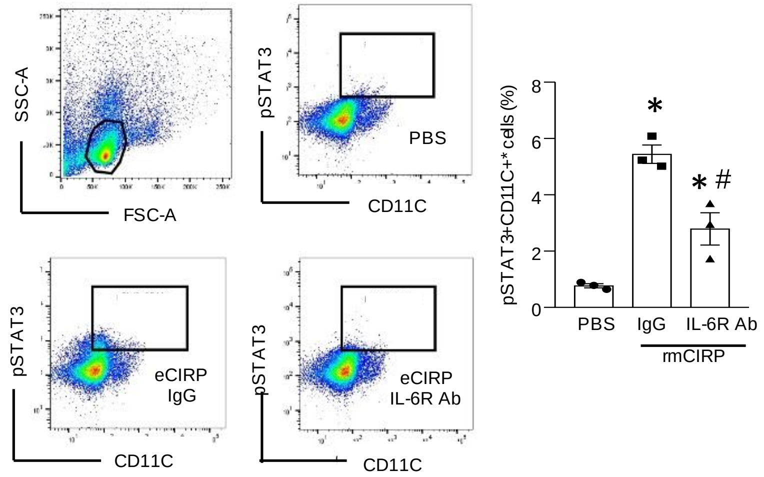

Supplemental Figure 9.

(Original Figure 4A repeated data) Peritoneal macrophages (1 × 10^6/ml) were stimulated with rmCIRP (1 µg/ml), and surface expression of IL-6R was assessed at 24 h following rmCIRP treatment by flow cytometry. Data is expressed as mean ± SE. The groups were compared by two-tailed Student's t-test (*P<0.05 vs. PBS). **(Original Figure 5A repeated data)** Splenocytes were pre-treated with IgG or anti-IL-6R Ab and stimulated with rmCIRP for 5 h. Cells were fixed, permeabilized, and stained with anti-pSTAT3 and CD11C Abs and analyzed. Data is expressed as mean ± SE. The groups were compared by one-way ANOVA and SNK method (*P<0.05 vs. PBS; #P<0.05 vs. IgG+rmCIRP). *Macrophages also express CD11C.

Supplemental Figure 10

Figure 5B (repeated experimental data)

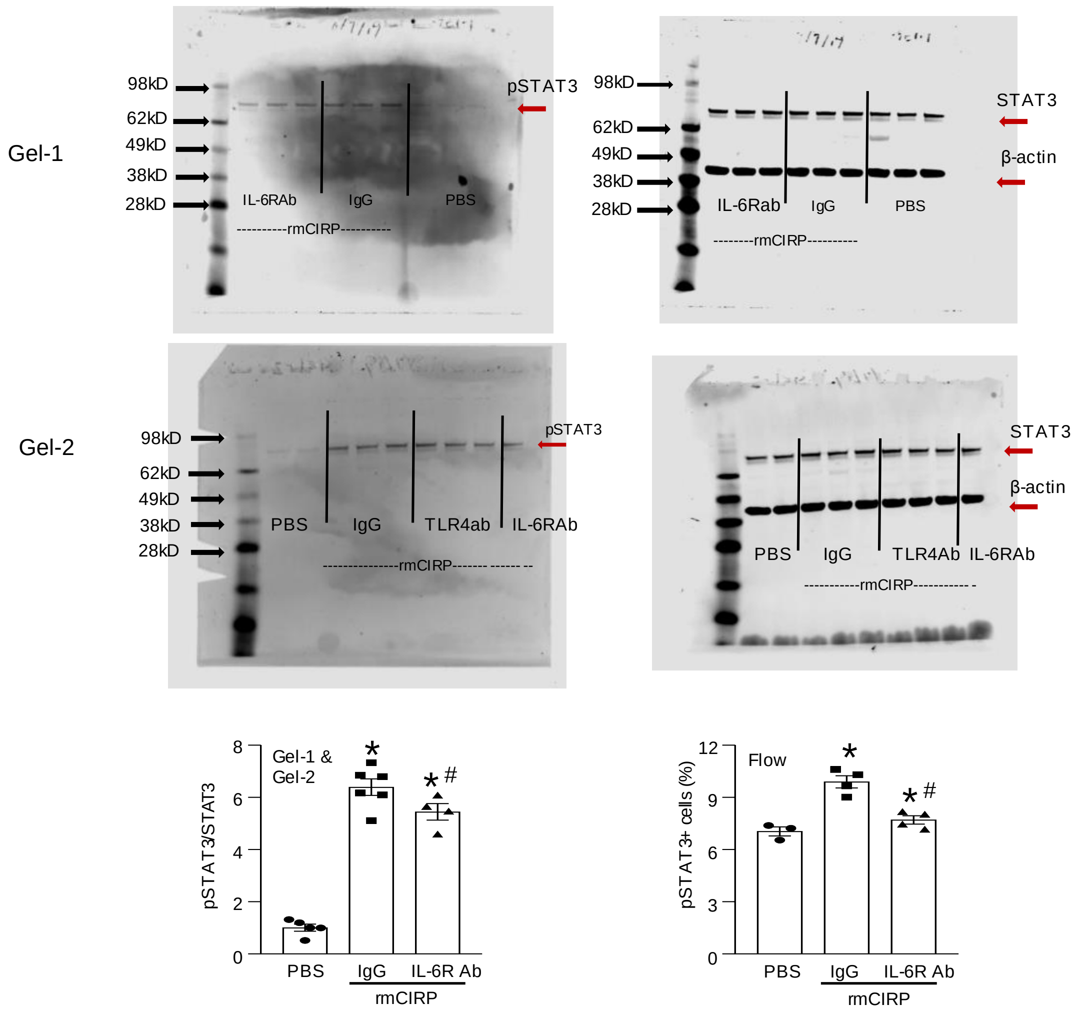

Supplemental Figure 10.

(Original Figure 5B repeated data) Peritoneal macrophages were pre-treated with IgG or anti-IL-6R Abs for 30 min, and stimulated with PBS or rmCIRP for 24 h. Total proteins were subjected to western blotting or flow cytometry using anti-pSTAT3, STAT3, and β-actin Abs. Data is expressed as mean ± SE. The groups were compared by one-way ANOVA and SNK method (*P<0.05 vs. PBS; #P<0.05 vs. IgG + rmCIRP).

Supplemental Figure 11

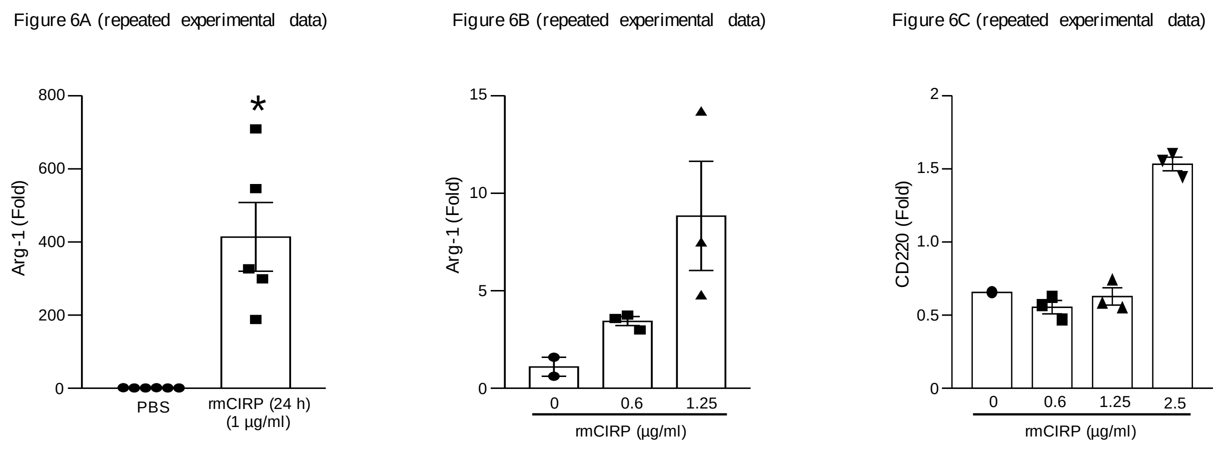

(Original Figure 6A-C repeated data) (A) RAW264.7 cells (1 × 10^6/ml) were treated with rmCIRP (1 µg/ml) for 24 h. Arg-1 expression at mRNA levels were assessed by qPCR. Expression of Arg-1 was normalized to β-actin expression and represented as fold induction compared to the normalized values of PBS control-treated cells. Data is expressed as mean ± SE. The groups were compared by two-tailed Student's t-test (**P*<0.05 vs. PBS). **(B, C)** RAW264.7 cells (1 × 10^6/ml) were treated with rmCIRP at dose of 0.625, 1.25, and 2.5 µg/ml for 48 h, the expression of Arg-1 and CD206 mRNAs were assessed by qPCR and normalized to β-actin expression. Results are represented as fold induction compared to the normalized values of PBS control-treated cells. Data is expressed as mean ± SE.

Supplemental Table 1: Primer sequences for real-time qPCR.

Gene	Forward Sequence	Reverse Sequence
TNF-α (X02611)	5' AGACCCTCACACTCAGATCATCTTC 3'	5' TTGCTACGACGTGGGCTACA 3'
Arginase-1 (NM_007482.3)	5' ATTATCGGAGCGCCTTTCTC 3'	5' GTGGTCTCTCACGTCATACT 3'
CD206 (NM_008625.2)	5' CCCAAGGGCTCTTCTAAAGCA 3'	5' CGCCGGCACCTATCACA 3'
β-actin (NM_007393)	5' CGTGAAAAGATGACCCAGATCA 3'	5' TGGTACGACCAGAGGCATACAG 3'
